# Potent and dose-sparing next-generation SARS-CoV-2 vaccine, mRNA-1283, induces polyfunctional and durable T cell immunity

**DOI:** 10.1038/s41541-026-01402-2

**Published:** 2026-02-19

**Authors:** Yamuna D. Paila, Rolando Pajon, Barbara Banbury, Paul Fields, Maha Maglinao, Stephen C. De Rosa, Daryl Morris, M. Juliana McElrath, Uma Siangphoe, Kristen W. Cohen, Robert Paris

**Affiliations:** 1 Moderna, Inc., Cambridge, MA 02142 USA; 2 Adaptive Biotechnologies, Seattle, WA 98109 USA; 3 Vaccine and Infectious Disease Division, Fred Hutchinson Cancer Center, Seattle, WA 98109 USA; 4 Department of Laboratory Medicine and Pathology, University of Washington School of Medicine, Seattle, WA 98195 USA

**Keywords:** Diseases, Immunology

## Abstract

Cell-mediated immunity contributes to durable protection against severe COVID-19, particularly as antibody responses wane or viral variants partially evade neutralization. Here, we characterize SARS-CoV-2-specific T cell responses elicited by a next-generation COVID-19 vaccine, mRNA-1283, which encodes the receptor-binding and N-terminal domains of the spike protein, in a phase 1 randomized clinical trial (NCT04813796). COVID-19-naïve, healthy adults (18–55 years) received two doses of mRNA-1283 (10 µg, 30 µg, or 100 µg), two doses of mRNA-1273 (100 µg; full-length spike comparator), or a single-dose regimen of mRNA-1283. Using intracellular cytokine staining, we show that two-dose regimens of mRNA-1283 or mRNA-1273 induce Th1-biased, polyfunctional spike-specific CD4+ and CD8+ T cell responses that were maintained through Day 209. TCRβ sequencing demonstrated significant increases in the breadth and frequency of SARS-CoV-2-associated TCRs, which correlated with functional spike-specific T cell responses. Therefore, the low-dose (10 µg) regimen of the next-generation COVID-19 vaccine, mRNA-1283, induces polyfunctional and durable CD4+ and CD8+ T cell immunity comparable to the standard 100 µg mRNA-1273 vaccine, supporting a dose-sparing strategy without compromising long-term cellular protection against severe COVID-19.

## Introduction

COVID-19 has resulted in substantial global morbidity and mortality^1^. Vaccination remains the most effective strategy to prevent severe disease and death caused by SARS-CoV-2 and its emergent variants^2^. Current COVID-19 vaccines elicit robust binding and neutralizing antibody responses that correlate with protection against symptomatic infection^3–5^. However, antibody responses wane over time and can be evaded by viral variants. In contrast, SARS-CoV-2-specific T cell responses have been associated with protection against severe disease, reduced hospitalization, and milder clinical outcomes following both infection and vaccination^6–8^. CD4+ and CD8+ T cells contribute to viral clearance, immune memory and cross-recognition of viral variants^9–12^, supporting durable protection even in the setting of reduced neutralizing antibody activity. Together, these observations highlight the complementary roles of humoral and cellular immunity in long-term protection against COVID-19 and underscore the importance of evaluating vaccine-induced T cell responses.

Vaccine-elicited cellular immunity is mediated primarily by CD4+ and CD8+ T cells, which coordinate complementary antiviral functions^13^. CD8+ cytotoxic T lymphocytes eliminate virus-infected cells and limit disease progression^14^, while CD4+ T helper (Th) cells provide critical immunoregulatory support, with Th1 responses promoting antiviral effector function and CD8+ T cell activity and Th2 responses supporting humoral immunity^15,16^. Together, these T cell subsets shape the magnitude, quality, and durability of vaccine-induced immune responses, providing a mechanistic basis for evaluating cellular immunity alongside antibody responses in COVID-19 vaccination strategies.

The original mRNA-1273 vaccine (Spikevax; Moderna, Inc., Cambridge, MA, USA^17^) encodes a prefusion stabilized full-length SARS-CoV-2 spike (S) protein^18^ and has demonstrated an acceptable safety profile with high clinical efficacy and real-world effectiveness against severe COVID-19 and death^18,19^. In addition to eliciting robust binding and neutralizing antibody responses^20^, mRNA-1273 induces durable SARS-CoV-2–specific CD4+ and CD8+ T cell responses^21–24^, including in immunocompromised populations^25,26^. Importantly, vaccine-induced T cell responses to mRNA-1273 have been shown to cross-recognize multiple SARS-CoV-2 variants, including omicron sublineages^11^.

Next-generation mRNA-based COVID-19 vaccines offer the opportunity to build upon the established mRNA-lipid nanoparticle (LNP) platform while addressing limitations related to dose, manufacturability and global distribution. The investigational mRNA-1283 vaccine encodes the receptor binding domain (RBD) and N-terminal domain (NTD) of the SARS-CoV-2 spike protein. By selectively expressing two highly immunogenic regions of spike using a shorter mRNA sequence, mRNA-1283 has demonstrated increased potency, eliciting comparable or higher humoral immune responses at lower dose levels (10 µg) than mRNA-1273 (100 µg) when administered as a primary vaccine series^27^ or as a booster^28^. mRNA-1283 has also demonstrated improved mRNA stability, with the potential for longer refrigerated shelf life than mRNA-1273^29^. Together, these features of mRNA-1283 may overcome logistical challenges that limit vaccine access and coverage globally^30^ and facilitate incorporation into combination respiratory vaccines, including the SARS-CoV-2/influenza virus vaccine (mRNA-1083; Moderna, Inc.) under clinical evaluation (NCT06097273)^31^.

While the safety, reactogenicity, and humoral immunogenicity of mRNA-1283 have been evaluated in phase 1 and 2 randomized clinical trials^27,28^, as well as in a recent phase 3 trial demonstrating higher efficacy and superior immunogenicity compared to mRNA-1273^32^, its ability to induce durable functional and clonally diverse SARS-CoV-2-specific T cell responses has not been comprehensively characterized. In particular, it remains unclear whether a dose-sparing regimen of mRNA-1283 can elicit CD4+ and CD8+ T cell responses comparable in magnitude, quality, durability and repertoire diversity to those induced by the licensed mRNA-1273 vaccine^21–24^.

In this study, we provide a detailed characterization of SARS-CoV-2 S-specific cellular immune responses induced by mRNA-1283 in a phase 1 randomized clinical trial, using complementary functional and repertoire-based approaches. We show that a two-dose, low-dose (10 µg) regimen of mRNA-1283 induces Th1-biased, polyfunctional CD4+ and CD8+ T cell responses that are durable through six months and comparable to those elicited by a two-dose 100 µg mRNA-1273 regimen^21–24^. T cell receptor (TCR) β sequencing demonstrates broad and diverse expansion of spike-specific T cell clones that correlates with functional responses measured by intracellular cytokine staining (ICS). Together, these findings establish that mRNA-1283 elicits durable and high-quality cellular immunity while supporting a dose-sparing vaccination strategy.

## Results

### Participants

**Fig. 1:**
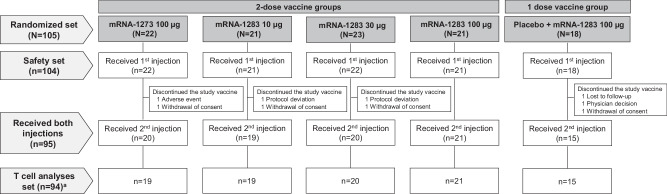
Participant disposition. ^a^Randomly assigned participants who received both doses of the study vaccination and had blood collected for cell-mediated immunity testing were included in the T cell analysis set.

A total of 105 SARS-CoV-2-naïve participants were randomized to receive two doses of mRNA-1283 (10 µg, 30 µg, 100 µg) or mRNA-1273 (100 µg), or a single dose of mRNA-1283 (100 µg) between March 11, 2021, and July 25, 2023. Among those randomized, 94 participants (two-dose regimens, *n* = 79; one-dose regimen, *n* = 15) were included in the analysis of T cell responses (Fig. 1). Baseline demographic characteristics were similar across groups; 56% of the participants were male, 72% were White, and 29% were Hispanic or Latino (Table S1).

### mRNA-1283 vaccination elicited Th1-biased CD4+ T cell responses targeting the S1 subunit

Spike-specific CD4+ and CD8+ T cell responses were assessed by ICS assay^33–35^, at baseline (Day 1) and on Days 36, 57, and 209. At baseline, low-frequency Th1 CD4+ T cell responses were detected in a small number of participants across vaccine groups. Since this population was SARS-CoV-2 naïve by serology, it is likely that these low-level responses reflect pre-existing cross-reactive T cell responses induced by endemic coronaviruses^36–38^.

**Fig. 2:**
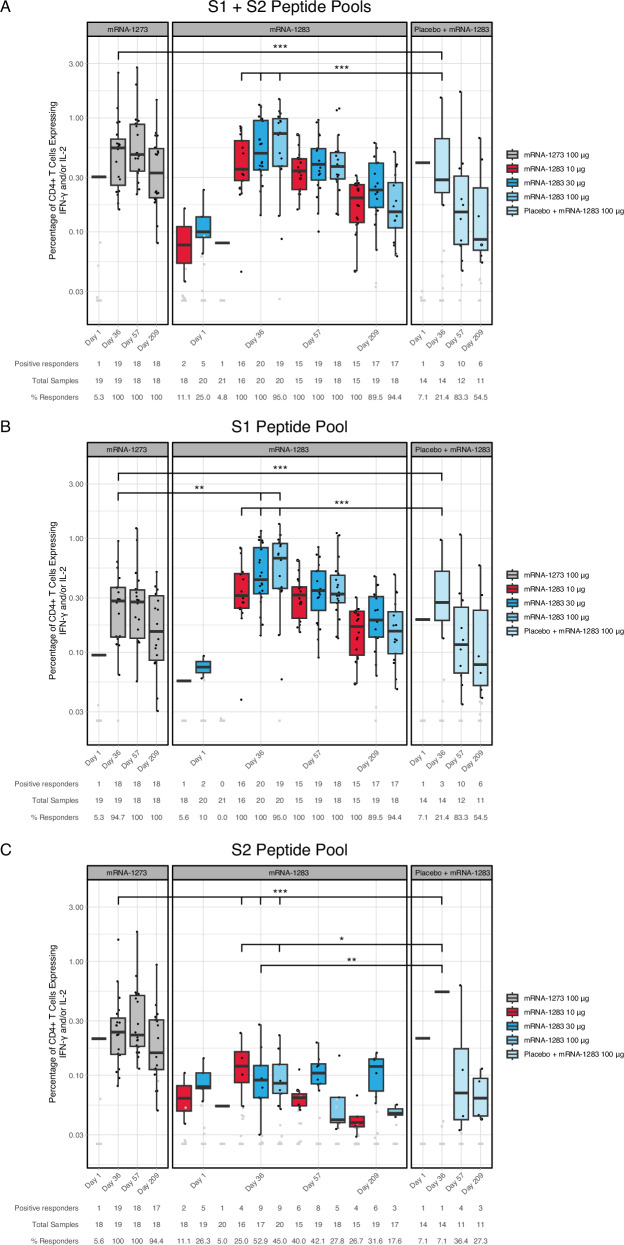
S-specific CD4+ Th1 T cell responses in study participants. Frequencies of CD4+ T cells expressing IFN-γ or IL-2 from cryopreserved PBMCs of mRNA-1283 or mRNA-1273 vaccine groups following ex vivo stimulation with SARS-CoV-2 peptides covering: (**A**) Total S, (**B**) S1, and (**C**) S2 at Visit 1 (baseline), Visit 4 (Day 36), Visit 5 (Day 57), and Visit 6 (Day 209) as measured by flow cytometry. Individual participant response data (represented by dots) are shown by vaccine group and time point. Black data points (dots) represent positive responses, while gray data points (dots) represent negative responses. Box plots display the distribution of positive responses; boxes and horizontal bars denote the first and third quartiles and the medians, respectively, and whisker endpoints equal to the largest value within 1.5 times the first and third quartiles. Statistical significance between vaccine groups at Day 36 are presented in the Figure. *P*-values were calculated by the Wilcoxon rank sum test. ***P < 0.0005, **P < 0.005, *P < 0.05. Table S4 provides details on the statistical significance between vaccine groups through Day 209, and Fig. S1 shows the schematic of the construct. IFN interferon, IL interleukin, PBMC peripheral blood mononuclear cell, S spike.

Following vaccination, all two-dose mRNA-1283 (10 µg and 30 µg) regimens and the mRNA-1273 regimen elicited S (S1 + S2) specific Th1 CD4+ T cell responses that peaked at Day 36 and were maintained through Day 209 in most participants (Fig. 2A; Table S2). In the single-dose mRNA-1283 regimen (dose 1: placebo, dose 2: mRNA-1283 100 µg), three of 14 participants (21.4%) and 10 of 12 participants (83.3%) had responses at Day 36 and Day 57, respectively (Fig. 2A**;** Table S2). The magnitude of S-specific Th1 responses induced by the 10-µg mRNA-1283 regimen was comparable to that induced by the 100-µg mRNA-1273 regimen at peak response (difference [confidence interval, CI]: 0.059 [−0.131 to 0.265]; *P* = 0.659; Wilcoxon rank sum test; Table S3). Consistent with the vaccine design, Th1 responses elicited by mRNA-1283 were predominantly directed against the S1 peptide pool, whereas responses following mRNA-1273 vaccination were more evenly distributed across S1 and S2 (Fig. 2B, C).

**Fig. 3:**
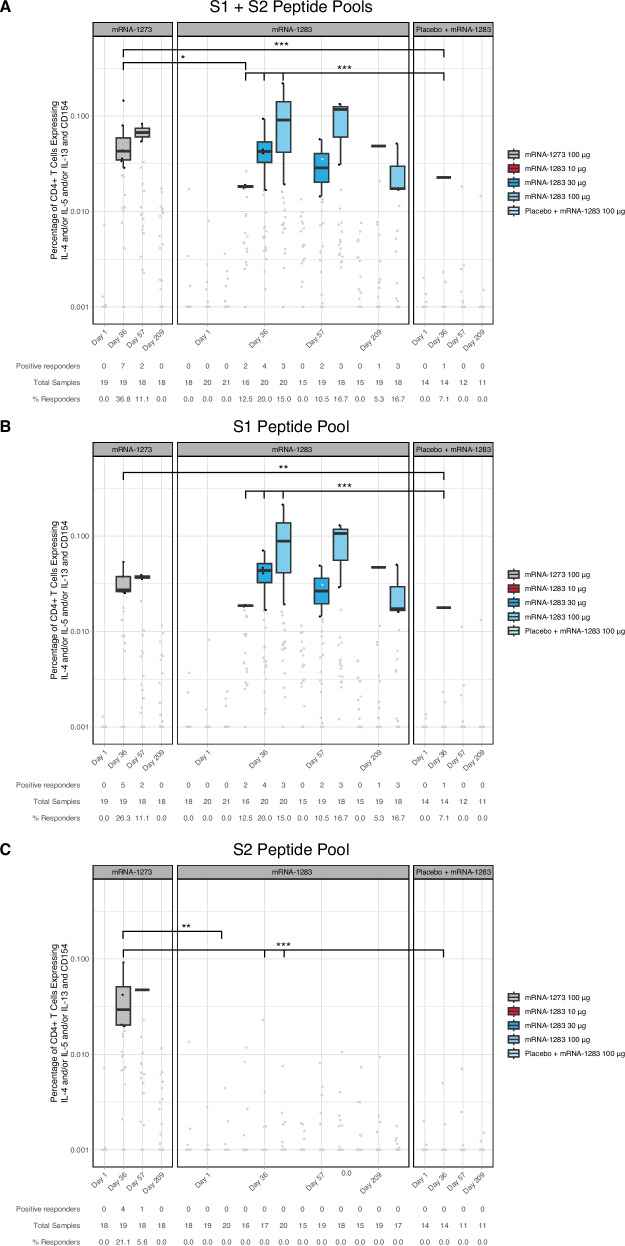
S-specific CD4+ Th2 T cell responses in study participants. Frequencies of CD4+ T cells expressing IL-4 and/or IL-5 and/or IL-13 and CD154 from cryopreserved PBMCs of mRNA-1283 or mRNA-1273 vaccine groups following ex vivo stimulation with SARS-CoV-2 peptides covering: (**A**) Total S, (**B**) S1, and (**C**) S2 at Visit 1 (baseline), Visit 4 (Day 36), Visit 5 (D57), and Visit 6 (D209) as measured by flow cytometry. Individual participant response data (represented by dots) are shown by vaccine group and time point. Black data points (dots) represent positive responses, while gray data points (dots) represent negative responses. Box plots display the distribution of positive responses; boxes and horizontal bars denote the first and third quartiles and the medians, respectively, and whisker endpoints equal the largest value within 1.5 times the first and third quartiles. Statistical significance between vaccine groups at Day 36 are presented in the Figure. *P*-values were calculated by the Wilcoxon rank sum test. ***P ≤ 0.0006, **P < 0.005, *P < 0.05. Table S5 provides details on the statistical significance between vaccine groups through Day 209, and Fig. S1 shows the schematic of the construct. IL interleukin, PBMC peripheral blood mononuclear cell, S spike.

S (S1 + S2) specific Th2 CD4+ T cell responses (expressing IL-4, IL-5, and/or IL-13 [or all 3 cytokines] with CD154) remained minimal across all vaccine groups. Although low-level increases were observed in some participants following vaccination, S-specific Th2 responses generally did not meet predefined criteria for statistical positivity and remained of low magnitude through Day 209 (maximum response rate of 37%, Fig. 3A–C**;** Table S2). Therefore, together with the above results, mRNA-1283 vaccination elicited a Th1-biased S-specific CD4+ T cell response, similar to mRNA-1273, as previously shown^21–24^.

### mRNA-1283 induced durable S-specific CD8+ T Cell responses

**Fig. 4:**
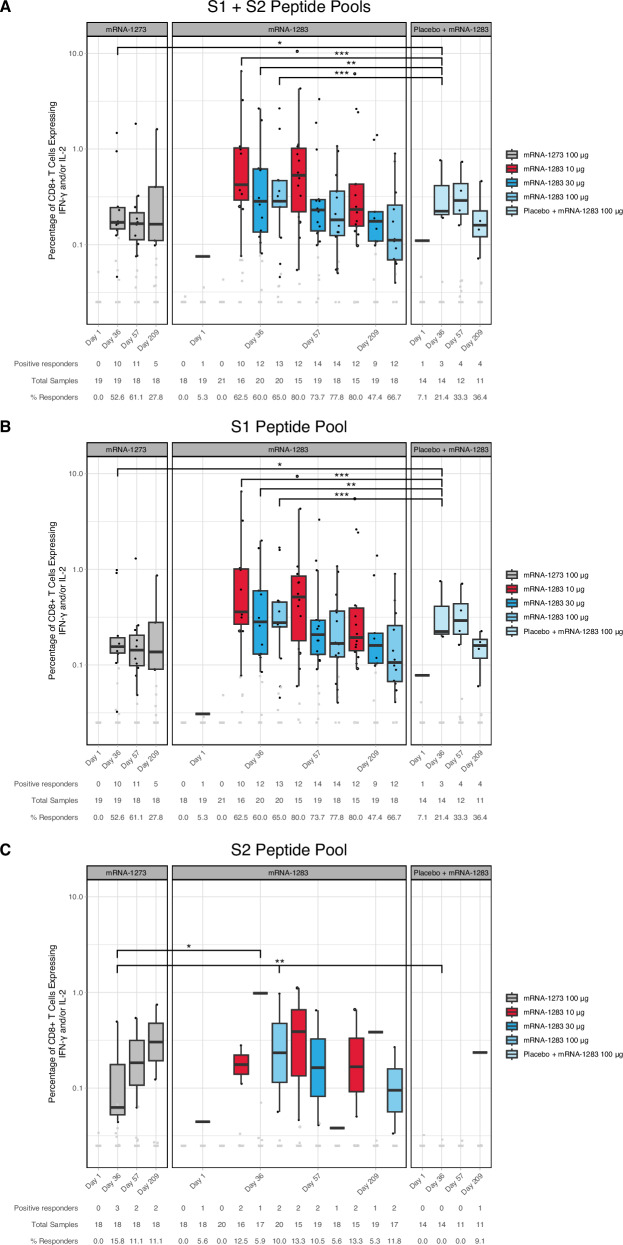
S-specific CD8+ T cell responses in study participants. Frequencies of CD8+ T cells expressing IFN-γ or IL-2 from cryopreserved PBMCs of mRNA-1283 or mRNA-1273 vaccine groups following ex vivo stimulation with SARS-CoV-2 peptides covering: (**A**) Total S, (**B**) S1, and (**C**) S2 at Visit 1 (baseline), Visit 4 (Day 36), Visit 5 (Day 57), and Visit 6 (Day 209) as measured by flow cytometry. Individual participant response data (represented by dots) are shown by vaccine group and time point. Black data points (dots) represent positive responses, while gray data points (dots) represent negative responses. Box plots display the distribution of positive responses; boxes and horizontal bars denote the first and third quartiles and the medians, respectively, and whisker endpoints equal the largest value within 1.5 times the first and third quartiles. Statistical significance between vaccine groups at Day 36 are presented in the Figure. *P*-values were calculated by the Wilcoxon rank sum test. ***P < 0.0005, ** < 0.005, * < 0.05. Table S6 provides details on the statistical significance between vaccine groups through Day 209, and Fig. S1 shows the schematic of the construct. IFN interferon, IL interleukin, PBMC peripheral blood mononuclear cell, S spike.

At baseline, S-specific CD8+ T cell responses, expressing IFN-γ and/or IL-2 upon stimulation with S1 and S2 peptide pools, were rarely detected across vaccine groups. Following vaccination, 61.1% to 80% of participants across two-dose regimens had significant S-specific CD8+ T cell responses by Day 57 that persisted through Day 209 (Fig. 4A**;** Table S2). Notably, the magnitude of S-specific CD8+ T cell responses was highest in participants receiving the 10 µg mRNA-1283 regimen at both Days 57 and 209 and was significantly greater than that observed following the 100 µg mRNA-1273 regimen (difference [CI]: Day 57, −0.261 [−0.588 to −0.007]; Day 209, −0.139 [−0.257 to −0.029]; *P* < 0.05; Wilcoxon rank sum test). For both the mRNA-1283 and mRNA-1273 vaccine groups, CD8+ T cell responses were predominantly directed against S1, with minimal responses to S2 (Fig. 4B, C).

### Vaccination-induced polyfunctional S-specific CD4+ and CD8+ T cells

The polyfunctionality of vaccine-induced T cells was assessed by measuring the simultaneous expression of key cytokines and effector molecules associated with antiviral immunity, including IFN-γ, IL-2, TNF-α, CD40L, granzyme B, and perforin. Both mRNA-1283 and mRNA-1273 induced highly polyfunctional S-specific CD4+ and CD8+ T cells (Figs. S2, S3). Among the 9 functional markers examined in CD4+ T cells, the majority of S-specific CD4+ T cells expressed combinations of up to 5 of these markers; cells co-expressing IL-17 were largely not detected among the responding cells. The most frequently detected functions included IFN-γ, IL-2, tumor necrosis factor-α (TNF-α), and CD40L, with granzyme B co-expression detected in some participants. Among the 9 functional markers examined in CD8+ T cells, S-specific CD8+ T cells most commonly expressed IFN-γ, IL-2, and TNF-α, and often co-expressed with either or both of the cytolytic molecules, granzyme B and perforin. Distribution of functional profiles was similar across all two-dose regimen vaccine groups and were maintained through Day 209 (Figs. S4, S5).

### Vaccination-induced durable S-specific memory CD4+ and CD8+ T Cells

Phenotypic analysis demonstrated that the majority (median of 65–76%) of S-specific CD4+ T cells exhibited an effector memory (EM; CD45RA− and CCR7−) phenotype one week after the second dose of mRNA-1283 or mRNA-1273, whereas 22–33% of S-specific CD4+ T cells had a central memory (CM; CD45RA− and CCR7+) phenotype (Fig. S6A). The distribution of memory CD4+ T cell populations was largely consistent across vaccine groups and through Day 209. Among S-specific CD8+ T cells at Day 36, 93–94% had an EM phenotype, with a small percentage (1–3%) exhibiting a TEMRA (CD45RA+ and CCR7−) or CM (2–5%) phenotype (Fig. S6B). By Day 209, the proportion of S-specific CD8+ T cells that were EM declined to 42–68%, with a concurrent rise in the TEMRA S-specific CD8+ T cells that peaked at Day 209 (24-50% TEMRA phenotype of S-specific CD8+ T cells); these longitudinal changes in memory phenotype were similar for the different vaccine arms.

### T cell receptor sequencing revealed SARS-CoV-2-specific TCR signatures and increased clonal breadth and frequency of SARS-CoV-2-specific TCR following vaccination

**Fig. 5:**
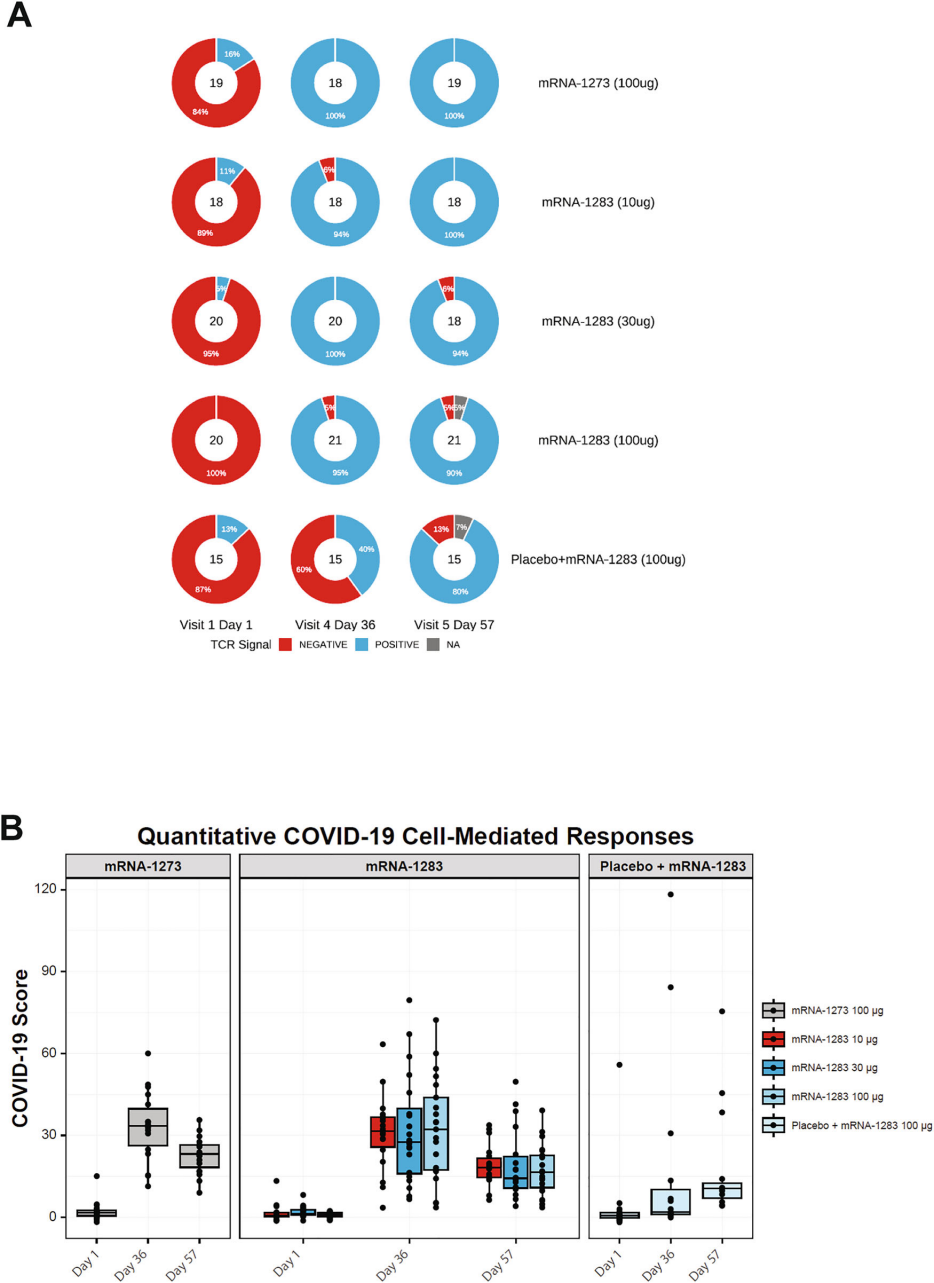
COVID-19–specific T cell responses by TCRβ sequencing. **A** Pie charts displaying COVID-19 classifier T cell signals (blue for a positive response, red for a negative response, and white for a missing sample at that visit) for participants according to vaccine group and time point. The classifier identified individuals with prior exposure to SARS-CoV-2 by detecting TCR*β* sequences that are commonly seen in COVID-positive or recently vaccinated individuals. Two samples are greyed, as they fall outside of the classifier trainer due to too many rearrangements. **B** COVID scores are shown for each individual participant (represented by dots) according to vaccine group and time point. Boxes and horizontal bars denote the first and third quartiles and the medians, respectively, and whisker endpoints equal the largest value within 1.5 times the first and third quartiles. Table S7 provides details on the statistical significance of COVID scores according to vaccine groups.

To complement the functional analyses of T cell responses to mRNA-1283 and mRNA-1273 vaccination, TCRβ sequencing was performed to quantify the presence and diversity of SARS-CoV-2-specific T cells in whole blood samples collected at baseline and following vaccination. The TCRβ repertoires were first analyzed using an approach that was developed to distinguish prior SARS-CoV-2 exposure, termed the COVID-19 classifier^39,40^. This classifier is based on the identification of SARS-CoV-2-specific TCRβ chains that have expanded after SARS-CoV-2 infection and/or vaccination and thus indicate the presence of SARS-CoV-2-specific T cell responses^39,40^. Most participants (81/92 [88.0%]) converted to positive for the COVID-19 T cell classifier by Day 36 (Fig. 5A), consistent with the induction of SARS-CoV-2-specific T cell responses. SARS-CoV-2-specific T cell responses were detected in 94.4% (17/18), 100%, and 95.2% (20/21) of participants receiving two doses of mRNA-1283 at 10-µg, 30-µg, and 100-µg dose levels, respectively, and in all participants receiving two doses of mRNA-1273. In contrast, responses were detected in 40.0% (6/15) of participants receiving a single-dose regimen of mRNA-1283. Of the eight participants with a positive baseline T cell response, one participant in the single-dose group transiently converted to negative at Day 36, with all eight participants positive again by Day 57.

The COVID-19 classifier incorporates a quantitative COVID score, defined as the log-odds (z-score) of the logistic regression model and reflecting deviation from pre–COVID-19 pandemic control repertoires (see Methods)^41^. All two-dose regimens of mRNA-1283 induced increases in COVID scores at Day 36 comparable to those observed with mRNA-1273 (Fig. 5B). COVID scores did not differ statistically between two-dose mRNA-1283 and mRNA-1273 groups, whereas scores for the single dose mRNA-1283 group were significantly lower at Day 36 (*P* < 0.05; Wilcoxon rank sum test), but increased by Day 57.

**Fig. 6:**
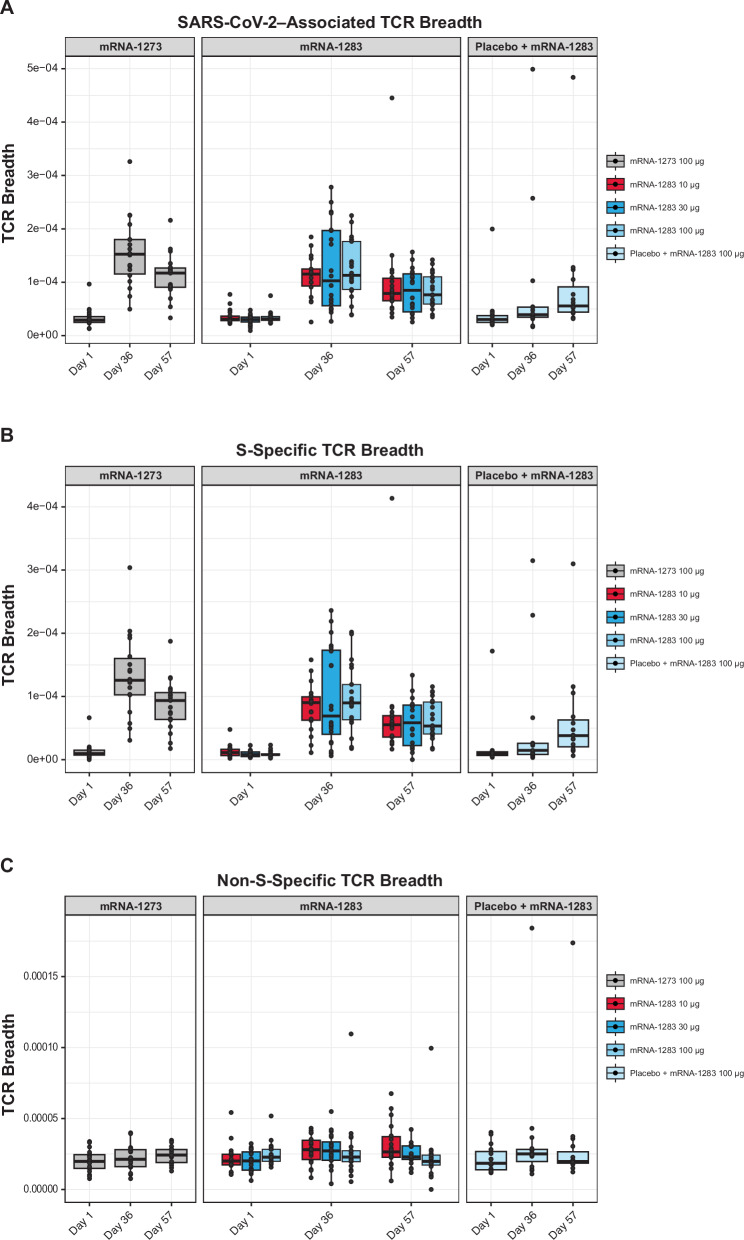
Breadth of total SARS-CoV-2, S-specific, and non-S-associated TCRs after vaccination. Each panel displays the number of (**A**) SARS-CoV-2–associated, (**B**) SARS-CoV-2 S-specific, or (**C**) SARS-CoV-2 non-S-specific unique TCRs relative to the total number of unique TCRs for each vaccine group and time point. Dots represent each individual participant’s repertoire. Boxes and horizontal bars denote the first and third quartiles and the medians, respectively, and whisker endpoints equal the largest value within 1.5 times the first and third quartiles. Statistical details are represented in Table S8. S spike, TCR T cell receptor.

The breadth and specificity of SARS-CoV-2-specific T cell responses were quantified by mapping participant CDR3 TCRβ sequences to a curated set of SARS-CoV-2-specific TCRs identified via Multiplex Identification of T cell Receptor Antigen specificity (MIRA) and enriched in COVID-19 cases relative to controls^39,42^. SARS-CoV-2-specific T cell responses were characterized by calculating the fraction of TCRs mapped to different epitopes (breadth) and the summed frequency of those TCRs. At Day 36, the breadth of SARS-CoV-2-associated TCRs increased significantly from baseline across all vaccine groups (all *P* < 0.05; Wilcoxon signed rank test; Fig. 6A). Breadth was similar between all two-dose mRNA-1283 and mRNA-1273 regimens, with the exception of higher breadth in the mRNA-1273 100-µg group compared with the mRNA-1283 10-µg group (*P* = 0.02; Wilcoxon rank sum test). Participants receiving a single dose of mRNA-1283 exhibited significantly lower breadth at Day 36 than all two-dose regimens (all *P* < 0.05). At Day 57, the breadth of SARS-CoV-2-associated TCRs remained stable for the two-dose mRNA-1283 and mRNA-1273 groups and increased modestly in the single-dose group.

A similar pattern was observed for S-specific TCR breadth (Fig. 6B) which increased significantly at Day 36 for all two-dose regimens (all *P* < 0.05; Wilcoxon signed rank test) and was higher than in the single-dose mRNA-1283 group (all *P* < 0.01; Wilcoxon rank sum test). S-specific breadth was comparable across two-dose vaccine groups, although higher in the mRNA-1273 100-µg group relative to the mRNA-1283 10-µg group (*P* = 0.007; Wilcoxon rank sum test). In contrast, the breadth of non-S-specific TCRs remained largely unchanged, with a significant increase observed only in the mRNA-1283 30-µg group (Fig. 6C; *P* = 0.02).

Vaccination also significantly increased the summed frequency of SARS-CoV-2-associated TCRs at Day 36 (all *P* < 0.01; Wilcoxon signed rank test), driven primarily by expansion of S-specific TCRs (Fig. S7). Frequencies of SARS-CoV-2-associated and S-specific TCRs were significantly higher in all two-dose regimens compared with the single-dose regimen (all *P* < 0.05; Wilcoxon signed rank test), whereas no significant differences were observed between the two-dose mRNA-1283 and mRNA-1273 groups.

**Fig. 7:**
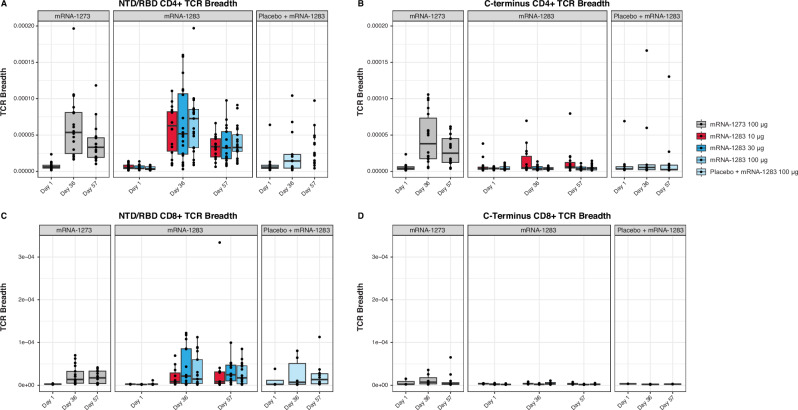
Epitope-level breadth of SARS-CoV-2 S-specific TCR repertoires following vaccination. Vaccine-specific differences in NTD/RBD- and C-terminal–directed SARS-CoV-2 spike TCR breadth. Breadth of S-specific NTD/RBD TCRs in (**A**) CD4+ and (**C**) CD8+ T cells or breadth of C-terminus TCRs in (**B**) CD4+ and (**D**) CD8+ T cells after vaccination. Each panel displays the breadth of individual participant repertoires (as represented by dots) according to vaccine group and time point. Boxes and horizontal bars denote the first and third quartiles and the medians, respectively, and whisker endpoints equal the largest value within 1.5 times the first and third quartiles. Statistical details are represented in Table S9. N-terminal domain; RBD receptor binding domain, S spike, TCR T cell receptor.

Epitope-level analyses demonstrated significant increases in the breadth of NTD/RBD (S1)-specific CD4+ TCRs at Day 36 for all two-dose regimens (all *P* < 0.05; Wilcoxon signed rank test; Fig. 7A), with no significant differences between the two-dose groups. In contrast, increased breadth of C-terminal (S2)-specific CD4+ TCRs was observed only in the mRNA-1273 100-µg group (*P* = 0.0001; Wilcoxon signed rank test; Fig. 7B), consistent with the full-length spike antigen encoded by mRNA-1273. Similarly, significant increases in the breadth of NTD/RBD-specific CD8+ TCRs at Day 36 were only observed in the two-dose regimens (*P* < 0.05; Wilcoxon signed rank test; Fig. 7C); whereas breadth of C-terminal-specific CD8+ TCRs did not increase significantly in any group (Fig. 7D).

Consistent with these findings, the summed frequencies of S-specific CD4+ TCRs increased significantly at Day 36 relative to baseline for all vaccine groups (all *P* < 0.05; Wilcoxon signed rank test; Fig. S8A), with higher frequencies observed for all two-dose regimens compared with the single-dose regimen (*P* < 0.05; Wilcoxon rank sum test). Conversely, significant increases in the summed frequencies of S-specific CD8+ TCRs were only observed following two-dose regimens (*P* < 0.05; Wilcoxon signed rank test; Fig. S8B).

### S-Specific TCRs correlated with functional T cell responses

The summed frequencies of S-specific CD4+ and CD8+ TCRs were compared with the frequencies of S-specific CD4+ and CD8+ T cell responses by ICS at baseline, Day 36, and Day 57 (Fig. S9). S-specific CD4+ T cell responses were significantly correlated at Day 36 (*r* = 0.59; *P* < 10^−5^) and Day 57 (*r* = 0.56; *P* < 10^−5^). S-specific CD8+ T cell responses were significantly correlated at Day 36 (*r* = 0.52; *P* < 10^−5^), but less so at Day 57 (*r* = 0.3*; P* = 0.019). At the epitope level, NTD/RBD-specific CD4+ T cell responses were significantly correlated between assays at Day 36 (r = 0.63; *P* < 10^−5^) and Day 57 (*r* = 0.47; *P* = 0.00002), whereas NTD/RBD-specific CD8+ responses were significantly correlated at Day 36 (*r* = 0.44; *P* = 0.00049) and less so at Day 57 (*r* = 0.31; *P* = 0.021). C-terminal–specific CD4+ T cell responses also showed significant correlations at Day 36 (*r* = 0.43; *P* = 0.00047) and Day 57 (*r* = 0.62; *P* < 10^−5^), whereas the C-terminal-specific CD8+ T cell responses were not significantly correlated at either time point (Day 36, *r* = 0.4; *P* = 0.077; Day 57, *r* = 0.16; *P* = 0.43).

### Conservation of T cell epitopes in omicron variant predicts cross-recognition

To assess the potential impact of SARS-CoV-2 omicron variant mutations on vaccine-elicited T cell responses, TCRβ sequences mapped to ancestral SARS-CoV-2 (Wuhan-Hu-1 strain) epitopes identified via MIRA were aligned with omicron spike mutations^43,44^. The predicted impact of omicron mutations on the CD4+ T cell epitope breadth was relatively low, with the dominant CD4+ T cell response spanning spike residues 160–218, and only including an omicron-specific mutation at approximately position 211–214 (Fig. S10A). Similarly, the predicted effects on CD8 + T cell epitope breadth were minimal, with few omicron mutations overlapping MIRA-defined spike epitopes (Fig. S10B), consistent with prior reports of preserved vaccine-induced cellular immunity to omicron variants^11^.

### Vaccination induced rapid expansion of unique T cell clones

**Fig. 8:**
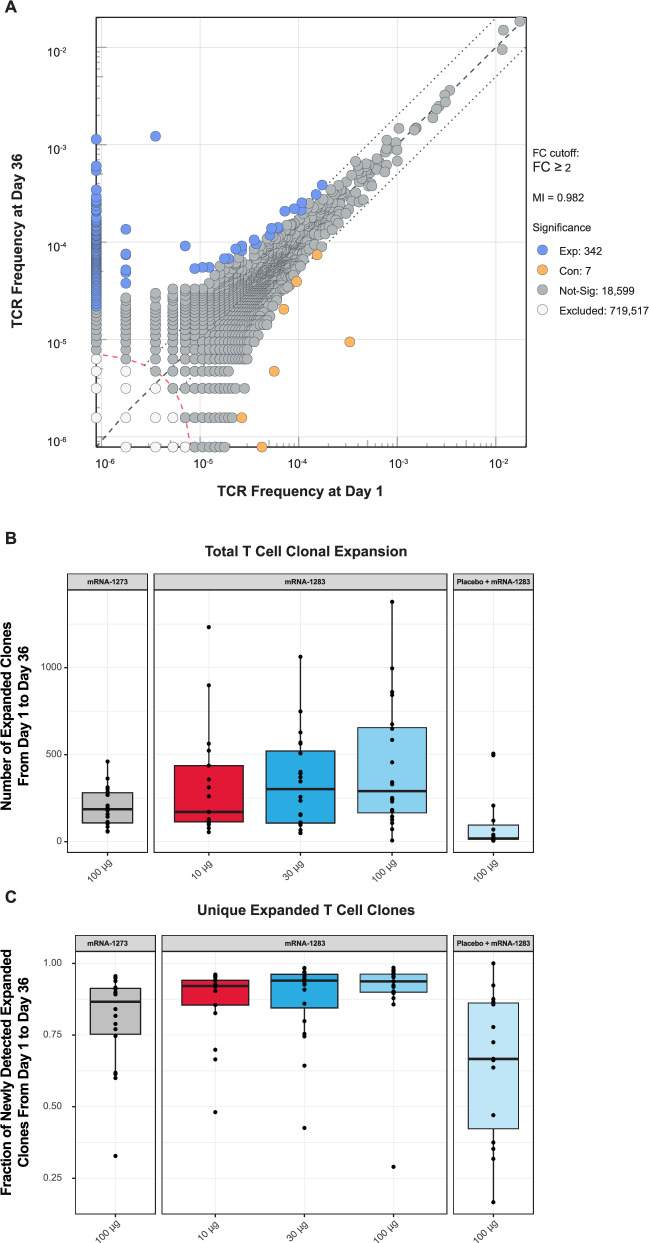
SARS-CoV-2 specific T cell clonal expansion after vaccination. **A** Scatterplot comparing TCR frequencies from a representative participant in the mRNA-1283 100-µg group at Day 1 (x-axis) and Day 36 (y-axis). Significantly differentially abundant clones are indicated in blue (expanded) and orange (contracted). Expanded and newly detected clones are blue points along the y-axis. The dashed line represents y = x (equivalent frequencies in both samples), while the dotted lines show the FC cutoff. The dashed red line demarcates the clones excluded from statistical testing due to low combined template count. **B** Number of expanded clones from Day 1 to Day 36 according to vaccine group, with boxes and horizontal bars denote the first and third quartiles and the medians, respectively, and whisker endpoints equal to the largest value within 1.5 times the first and third quartiles. **C** Fraction of newly detected expanded clones from DaCy 1 to Day 36 according to vaccine group. Boxes and horizontal bars denote the first and third quartiles and the medians, respectively, and whisker endpoints equal to the largest value within 1.5 times the first and third quartiles. Table S10 provides statistical details on T cell clonal expansion after vaccination. FC fold change, S spike, TCR T cell receptor.

T cell clonal expansion after vaccination was examined by identifying differentially abundant TCRβ clones after two-dose regimen mRNA-1283 and mRNA-1273 regimens at Day 36; however, all two-dose regimens exhibited significantly greater clonal expansion than the single-dose mRNA-1283. Representative clonal expansion is shown in Fig. 8A, with analyses focused on the baseline to Day 36, corresponding to the peak clonal expansion across regimens (Fig. S11). No significant differences in the number of expanded clones were observed between two-dose mRNA-1283 and mRNA-1273 regimens at Day 36. However, all two-dose regimens had a significantly higher number of expanded clones than the single-dose mRNA-1283 regimen (all *P* < 0.05; Wilcoxon rank sum test; Fig. 8B). Although median numbers of expanded clones were numerically higher in the mRNA-1283 30 µg and 100 µg groups compared with mRNA-1273 100 µg, these differences were not statistically significant. In the two-dose regimens, more than 75% of all newly expanded clones between baseline and Day 36 were unique, which was significantly higher diversity than observed in the single-dose mRNA-1283 group (all *P* < 0.05; Wilcoxon rank sum test; Fig. 8C).

## Discussion

This study provides a comprehensive characterization of cellular immune responses elicited by the next-generation COVID-19 vaccine, mRNA-1283, as a primary series in healthy adults without prior SARS-CoV-2 infection. Using complementary functional and repertoire-based approaches, we demonstrate that a two-dose, low-dose (10 µg) regimen of mRNA-1283 induces durable, polyfunctional S-specific CD4+ and CD8+ T cell responses comparable to those elicited by the licensed mRNA-1273 vaccine. These findings extend prior reports on mRNA-1283 that focused primarily on safety, reactogenicity, and humoral immunogenicity^27,28,32^, by providing the first detailed longitudinal assessment of T cell polyfunctionality, memory differentiation, and TCR repertoire diversity in the context of antigen minimization and dose sparing.

Across all two-dose regimens, mRNA-1283 elicited predominantly Th1-biased CD4+ T cell responses with minimal Th2 polarization, consistent with immune profiles previously observed following mRNA-1273 vaccination^21–24^. S-specific CD8+ T cell responses were also durable through six months, with higher magnitudes observed in participants receiving the 10-µg mRNA-1283 regimen. This was observed despite the fact that 10 μg of mRNA-1283 contains fewer mRNA-LNP molecules than a 100 μg dose of mRNA-1273 (0.17 vs 0.71 nmol). While definitive mechanistic conclusions cannot be drawn from this study, this finding may reflect higher in vivo protein expression, as suggested by preclinical assessments in which mRNA-1283 induced greater cell-surface expression of RBD and NTD in vitro in transfected HEK293T cells and in vivo in antigen-presenting cells within lymph nodes of injected mice compared with mRNA-1273^45^.

Importantly, both CD4+ and CD8+ T cell responses were polyfunctional, with individual cells co-expressing multiple cytokines and effector molecules associated with antiviral activity, including IFN-γ, IL-2, TNF-α, CD40L, granzyme B, and perforin. Polyfunctional T cells have been associated with enhanced effector capacity, proliferative potential, and long-term persistence, supporting the quality and durability of the vaccine-elicited cellular immune responses^46–48^. Longitudinal phenotypic analyses further revealed distinct memory trajectories for CD4+ and CD8+ T cells. S-specific CD4+ T cell memory phenotypes remained relatively stable over time, consistent with maintenance of helper function. In contrast, S-specific CD8+ T cells showed a progressive increase in the TEMRA subset by Day 209, a phenotype associated with long-lived cytotoxic memory observed after effective antiviral vaccination^49^.

TCRβ repertoire sequencing complemented the functional analyses by providing insight into the breadth, specificity, and clonal dynamics of vaccine-induced T cell responses, with two-dose regimens of mRNA-1283 inducing significant expansions of SARS-CoV-2-associated and S-restricted TCRs with breadth, frequency and diversity comparable to mRNA-1273 (100 µg). Summed frequencies of S-specific TCRs correlated with functional CD4+ and CD8+ T cell responses measured by intracellular cytokine staining, supporting the concordance between the repertoire-based and functional assessments of cellular immunity. At the epitope level, mRNA-1283 elicited T cell responses were predominantly directed against the NTD and RBD regions of the spike protein, consistent with the encoded antigen. In contrast, mRNA-1273 induced broader CD4+ TCR recognition across both S1 and S2 regions, reflecting its full-length spike construct. Despite this difference in immunofocusing, overall clonal diversity was similar between vaccines, suggesting that mRNA-1283 induces a polyclonal response within its targeted antigenic regions.

While S1-directed responses raise theoretical considerations regarding variant cross-recognition due to the higher mutational burden in this region, in silico analyses indicated that a substantial proportion of vaccine-elicited T cell epitopes were conserved across omicron sublineages^43,44^. These findings are consistent with prior studies demonstrating preservation of vaccine-induced T cell responses to SARS-CoV-2 variants despite reduced neutralizing antibody activity^11,50–53^. Nevertheless, computational predictions do not fully capture the complexity of antigen processing, epitope presentation, or TCR-peptide-MHC interactions^54,55^, and assessment of functional cross-reactivity against variant-specific peptide pools remains an important area for future investigation. In this context, the expansion and persistence of a diverse, S-specific TCR repertoire following mRNA-1283 vaccination, together with the ability to update vaccine strain composition^56^, supports the potential for maintaining cellular immune memory in the setting of ongoing viral evolution.

Several strengths of this study warrant consideration, including the randomized design with comparison to a licensed mRNA-based SARS-CoV-2 vaccine (mRNA-1273 [Spikevax; Moderna, Inc., Cambridge, MA])^17^, and the use of complementary methodologies to assess cellular immunity. The combined application of ICS and TCRβ sequencing enabled integrated evaluation of T cell magnitude, functional quality, memory phenotype, and clonal diversity following mRNA-1283 vaccination. While ICS and TCRβ sequencing yielded correlated measures of S-specific CD4+ and CD8 + T cell responses, these approaches are complementary rather than interchangeable. TCRβ sequencing does not capture functional attributes such as cytokine production or polyfunctionality, which remain uniquely accessible through ICS, but offers practical advantages for scalable immune monitoring in large, multi-site clinical trials. Some discrepancies between assays were observed, likely reflecting methodological and temporal differences rather than discordant biological responses. Importantly, ICS analyses remain essential for detailed functional and phenotypic assessment of vaccine-induced T cell responses, including polyfunctionality and memory differentiation^57^.

Limitations of this study include the relatively small sample size which limits statistical power and may not capture the full heterogeneity of vaccine responses. In addition, the TCRβ COVID-19 classifier was primarily trained on repertoires derived from individuals following primary SARS-CoV-2 infection and may underrepresent vaccine-elicited S-specific CD8+ T cell responses^33,58,59^. Reduced sensitivity for vaccine-elicited S-specific CD8+ TCRs could contribute to the lower correlation between ICS and TCRβ-based enumeration of S-specific CD8+ T cells. Furthermore, epitope assignments inferred from repertoire analyses cannot exclude overlapping specificities without fine epitope mapping. Finally, definitive correlates or protective thresholds for vaccine-induced T cell immunity have not been established; however, associations between the magnitude and quality of T cell responses and improved clinical outcomes have been demonstrated in SARS-CoV-2 infection studies^60–62^, and provide important context for these findings.

In conclusion, a two-dose regimen of mRNA-1283, including a low-dose (10 µg) formulation, induces durable SARS-CoV-2-specific CD4+ and CD8+ T cell responses comparable to those elicited by mRNA-1273 (100 µg). Together with previously reported humoral immunogenicity and efficacy data^27,28,32^, these findings support mRNA-1283 as a dose-sparing, next-generation COVID-19 vaccine capable of inducing durable antiviral cellular immunity.

## Methods

### Study design and participants

This analysis is part of a phase 1, randomized, observer-blinded study conducted at 5 US sites evaluating the safety, reactogenicity, and immunogenicity of mRNA-1283 in healthy adults aged 18 to 55 years (NCT04813796). A full description of the trial design has been previously described^27^. In brief, participants eligible for enrollment were healthy adults (aged 18-55 years) who had not previously received a COVID-19 vaccine, had a negative SARS-CoV-2 serology (anti-nucleocapsid antibodies), and had no known medical history of COVID-19 or recent (≤30 days) SARS-CoV-2 exposure. Eligible participants were randomly assigned in parallel (1:1:1:1:1) using interactive response technology to receive a two-dose regimen (28 days apart) of mRNA-1283 at 3 dose levels (10 µg, 30 µg, and 100 µg), a two-dose regimen (28 days apart) of mRNA-1273 100 µg, or a single-dose regimen of mRNA-1283 (placebo + mRNA-1283 100 µg). For this observer-blind study, participants, staff involved in participant assessment, and sponsor personnel were blinded to the participant-specific dosing assignment until the end of the study. Vaccine preparation and administration were facilitated by an unblinded staff member with no role in the observation or assessment of study participants.

### Vaccines

mRNA-1283 is an investigational LNP-encapsulated mRNA vaccine containing an mRNA sequence encoding the SARS-CoV-2 S-protein RBD and NTD, connected via a flexible peptide linker and anchored using a 23-amino acid influenza hemagglutinin transmembrane domain. mRNA-1273 is an mRNA-LNP vaccine encoding the full-length, prefusion-stabilized SARS-CoV-2 S protein. Placebo was a 0.9% sodium chloride (0.9% saline) injection. Vaccines were provided as a sterile liquid for injection and injected into the deltoid muscle at a final volume of 0.5 mL. mRNA-1283 was administered either as a two-dose regimen, 28 days apart (Day 1 and Day 29), or as a single-dose regimen with placebo administered on Day 1 and a single dose of mRNA-1283 (100 µg) administered on Day 29. mRNA-1273 100 µg was administered as a two-dose regimen, 28 days apart (Day 1 and Day 29).

### Objectives and endpoints

The findings for the primary and secondary objectives detailing the safety, reactogenicity, and humoral immunogenicity for this trial are reported separately^27^. This manuscript reports on an exploratory objective of the trial to assess SARS-CoV-2 S-specific cell-mediated immune responses. Endpoints included the identification of SARS-CoV-2 S-specific cellular immune responses.

### PBMC sample processing

Blood samples for cell-mediated immunogenicity analyses were collected on Day 1 (baseline), Day 36, Day 57, and Day 209 (Month 7). Peripheral blood mononuclear cells (PBMCs) were isolated and cryopreserved from whole blood collected in K2-EDTA tubes at clinical sites within 6 to 8 h of venipuncture using the standard PBMC isolation procedure^35,63,64^. PBMCs were cryopreserved in freezing media (heat-inactivated fetal bovine serum [FBS, Sigma-Aldrich] containing 10% dimethyl sulfoxide) and stored in liquid nitrogen until use. PBMCs were thawed and rested overnight at 37 °C; 5% CO_2_ in R10 (RPMI 1640 [GibcoBRL, NY, USA] containing 10% fetal calf serum [FCS; Gemini Bioproducts, CA], 2 mM L-glutamine [GibcoBRL], 100 U/mL penicillin G, and 100 μg/mL streptomycin sulfate) prior to stimulation. An arbitrary threshold of 66% minimum cell viability after overnight resting on the day following thaw was required for use in ICS assays^35^.

### Intracellular cytokine staining assay

SARS-CoV-2 Spike-specific CD4+ and CD8+ T-cell responses were evaluated using a high-dimensional, multi-parameter, validated ICS assay with a 27-color flow cytometry panel (Fig. S12)^33,34^. After thaw and rest, PBMCs were stimulated ex vivo with pooled peptides covering the SARS-CoV-2 D614G S subunit 1 (S1; comprising NTD and RBD domains) and the S subunit 2 (S2; Fig. S1) (final concentration of 1 µg/mL per 15-mer peptide, overlapping by 11 amino acids; Bio-Synthesis Inc., Lewisville, TX), in the presence of the secretory inhibitor, brefeldin A (10 µg/mL; Sigma-Aldrich, St. Louis, MO) and the co-stimulatory antibodies, CD28 and CD49d (each at 1 µg/mL; Becton Dickinson Biosciences, San Jose, CA). Fluorescently-labeled CD154 antibody (BD Biosciences, San Diego, CA) was also included in the stimulation cocktail. Stimulation with staphylococcal enterotoxin B (0.25 µg/ml; Sigma-Aldrich) served as a positive control, and the peptide diluent (0.5% dimethyl sulfoxide) served as the negative control. Cells were incubated for 6 hours at 37 °C maintained with 5% CO_2_. After the incubation, 20 µL 20 mM EDTA 1 × PBS was added to each well and the plates were transferred to 4 °C overnight. The next day, cells were washed and stained with viability dye (Thermo Fisher, Waltham, MA) in 1 × PBS for 20 min in the dark. The cells were washed again and incubated with the surface staining antibodies for 20 min in the dark. Subsequently, cells were fixed (BD FACS Lyse; BD Biosciences) for 10 min, centrifuged and permeabilized (BD FACS Perm II; BD Biosciences) for 10 min. The cytokine ICS was performed in the dark for 30 min, washed two times and resuspended in 1% paraformaldehyde 1 x PBS. Cells were acquired the same day on a custom LSRII (BD Biosciences).

S-specific T cells were identified, quantified, and characterized by upregulation of expressed cytokines and other effector molecules by multiparameter flow cytometry (Fig. S12). S-specific Th1 responses were characterized as live, CD14-, FSC_low_ SSC_low_, singlet, CD19-, CD16-, CD3+, and CD4+ T cells that expressed IFN-γ and/or IL-2, without coexpression of IL-4, IL-5, or IL-13. S-specific Th2 responses were defined as live, CD14-, FSC_low_ SSC_low_, singlet, CD19-, CD16-, CD3+CD4+ lymphocytes that expressed IL-4, IL-5, and/or IL-13 (or all 3 cytokines), with co-expression of CD154^65^. S-specific CD8+ T cell responses were defined as live, CD14-, FSC_low_ SSC_low_, singlet, CD19-, CD16-, CD3+, and CD8+ T cells that expressed IFN-γ, and/or IL-2. Cytokine-positive S-specific CD4+ memory T cell populations were further characterized as naïve (CD45RA+CCR7+), effector memory (CD45RA- CCR7-), and central memory (CD45RA− CCR7+). Cytokine-positive S-specific CD8+ memory T cell populations were characterized as naïve (CD45RA+CCR7+), effector memory (CD45RA− CCR7−), and TEMRA (CD45RA+CCR7−).

S-specific T cell responses to the S1 and S2 subunits were assessed both separately and combined for the overall response to the SARS-CoV-2 S peptides. Statistically positive S-specific T cell responses were determined using Fisher’s exact test by comparing the number of cytokine-positive CD4+ or CD8+ T cells out of total CD4+ or CD8+ T cells, respectively, observed in the peptide-stimulated condition versus the cytokine-positive cell number out of total CD4+ or CD8+ T cells observed in the negative control condition^26,33,35^. All ICS assays were conducted in a blinded manner. Polyfunctionality was calculated as the frequency of the Boolean combination of cytokines detected or not detected within the total CD4+ or CD8+ T cell population.

### Computational modeling of general SARS-CoV-2 T cell responses

All samples were classified as positive or negative for detection and enrichment of COVID-specific T cells using Adaptive’s COVID classifiers^39^. We employed the v3 classifier for this work, which was trained by comparing peripheral repertoires from 2408 COVID positive and recently vaccinated individuals (cases) with 3909 control samples collected before the COVID-19 pandemic. All classifiers were trained and validated on Adaptive in-house samples. In brief, TCR repertoires from cases and controls were compared using a 1-tailed Fisher exact test to identify public TCRβ sequences that were significantly enriched in cases. The final set of case-enriched sequences was used to train a simple 2-feature logistic regression classifier to predict previous exposure to SARS-CoV-2 antigens. We applied the final v3 classifier model to all samples in this study to get a predictive z-score (v3 COVID score) that describes the magnitude of enhanced sequences detected in the repertoire compared with the control population. Samples were then classified as positive or negative based on their scores, where the threshold for positivity was previously determined at 99.8% specificity. Previous application of these classifiers on Adaptive validation datasets has indicated high sensitivity (>97%) on exposure history, and COVID scores at the time of infection correlate with disease severity^39–41^.

### TCR variable beta chain sequencing

Immunosequencing of the complementarity-determining region 3 (CDR3) regions of human TCRβ chains was performed using TCRβ sequencing (Adaptive Biotechnologies, Seattle, WA). Extracted genomic DNA (isolated from PBMC samples collected on Day 1, Day 36, and Day 57) was amplified in a bias-controlled multiplex polymerase chain reaction, followed by high-throughput sequencing. Sequences were collapsed and filtered in order to identify and quantify the absolute abundance of each unique TCRβ CDR3 region for further analysis as previously described^66–68^.

### Mapping TCRβ sequences to SARS-CoV-2 antigens

TCR sequences were mapped against a set of sequences that are known to react to SARS-CoV-2. Briefly, these sequences were first identified by MIRA^42^, following allocation to antigen-specific addresses, which detail major histocompatibility complex class presentation and were used to correlate response with viral open reading frames and cellular subsets (CD8+ T cells vs CD4+ T cells). Reactive TCRs were further screened for enrichment in a repertoire previously infected with COVID-19 compared with repertoires that were negative for COVID-19 collected as part of ImmuneCODE, a publicly available open database, to remove TCRs that may be very frequent or cross-reactive to common antigens. Responses were quantified by the number and/or frequency of COVID-19–specific TCRs. Specifically, clonal breadth and sum frequency were determined for each sample. These 2 metrics were previously shown to provide good separation of cases (defined as either COVID-19 infected or vaccinated) and controls^39,69,70^.

### Comparison to known variants

Sequences of known variants were obtained from GISAID on December 1, 2021 (www.gisaid.org) and were aligned to known MIRA antigen locations. Antigens that contain any omicron mutations (identified based on mutations observed in more than 75% of published sequences from 172 individuals with COVID-19 infections of the omicron variant) were labeled as potentially impacted.

### Clonal expansion

Clonal expansion was calculated according to a binomial distribution framework, as described previously^71^. In brief, the combined count for each TCR was treated as a fixed number of trials (Bernoulli experiments) in a 2-sided binomial test. This test provided the probability, ***p***, of the observed template counts in each sample under the null hypothesis that these templates are evenly distributed between the 2 samples, relative to their respective repertoire sizes (ie, the total productive template count of each sample). A TCR is considered differentially abundant if *P* < 0.01 after applying the Benjamini-Hochberg procedure to control the false discovery rate (FDR)^72^ and the TCR at least doubled in its frequency (>2 log fold change).

### Statistics

This manuscript reports findings from post-hoc analysis of an exploratory objective of the trial. All statistical analyses conducted were thus exploratory in nature and not pre-specified in the study protocol. Statistical testing of differences between treatment groups or timepoints was not adjusted for multiple comparisons. All statistical comparisons of TCRβ metrics between arms were conducted using nonparametric Wilcoxon rank sum tests with no multiplicity adjustment for the statistical comparisons. Longitudinal changes in T cell responses from baseline to Day 36 were conducted using the Wilcoxon signed rank test. All statistical tests were conducted using R v4.3.x.

### Study approval

Written informed consent was provided by all participants prior to the initiation of study procedures. All study materials, including the protocol, were approved by the central institutional review board (Advarra, Inc. Columbia, MD, 21044, USA) and the institutional review board for each participating site. The study was conducted in accordance with the protocol, applicable laws and regulatory requirements, the International Council for Harmonization Good Clinical Practice guidelines, and the ethical principles derived from the Declaration of Helsinki and Council for International Organizations of Medical Sciences International Ethical Guidelines.

## Supplementary information

Supplementary information

Supplementary information

## Data Availability

Access to patient-level data presented in this article and supporting clinical documents with external researchers who provide methodologically sound scientific proposals will be available upon reasonable request for products or indications that have been approved by regulators in the relevant markets and subject to review from 24 months after study completion. Such requests can be made to Moderna Inc., 325 Binney Street, Cambridge, MA 02142, data_sharing@modernatx.com. A materials transfer and/or data access agreement with the sponsor will be required for accessing shared data. All other relevant data are presented in the paper. The protocol is available online at ClinicalTrials.gov: NCT04813796.
