# Supplementary material for: Potent and dose-sparing next-generation SARS-CoV-2 vaccine, mRNA-1283, induces polyfunctional and durable T cell immunity
Source: NPJ Vaccines. 2026 Feb 19;11:74. doi: 10.1038/s41541-026-01402-2 (PMC13031613; doi:10.1038/s41541-026-01402-2)
Supplement: Supplementary file 1 — Supplementary information [file 41541_2026_1402_MOESM1_ESM.pdf]

SUPPLEMENTARY INFORMATION

Table S1. Participant baseline demographics

	mRNA-1273	mRNA-1283			Placebo + mRNA-1283
	100 µg (n = 19)	10 µg (n = 19)	30 µg (n = 20)	100 µg (n = 21)	100 µg (n = 15)
Age, y					
Mean ± SD	42.3 ± 10.0	37.7 ± 9.5	34.2 ± 10.0	34.8 ±10.8	34.4 ± 9.9
Median (IQR)	43.0 (35-52)	41.0 (27-45)	30.5 (26-41.5)	36.0 (27-47)	31.0 (25-43)
Sex, n (%)					
Male	12 (63.2)	8 (42.1)	12 (60.0)	12 (57.1)	9 (60.0)
Female	7 (36.8)	11 (57.9)	8 (40.0)	9 (42.9)	6 (40.0)
Race, n (%)					
White	15 (79.0)	14 (73.7)	14 (70.0)	16 (76.2)	9 (60.0)
Black/African American	1 (5.3)	0	2 (10.0)	1 (4.8)	0
Asian	0	0	1 (5.0)	2 (9.5)	1 (6.7)
Native Hawaiian or Other Pacific Islander	0	0	0	0	1 (6.7)
Multiracial	0	1 (5.3)	0	0	2 (13.3)
Not reported	3 (15.8)	4 (21.1)	3 (15.0)	2 (9.5)	2 (13.3)
Ethnicity, n (%)					
Hispanic or Latino	6 (31.6)	7 (36.8)	7 (35.0)	5 (23.8)	2 (13.3)
Not Hispanic or Latino	13 (68.4)	12 (63.2)	13 (65.0)	16 (76.2)	12 (80.0)
Not reported	0	0	0	0	1 (6.7)
Weight, kg					
Mean ± SD	81.2 ± 12.3	80.2 ± 14.6	75.3 ± 16.9	73.4 ± 15.5	82.4 ± 21.7
Height, cm					
Mean ± SD	172.4 ± 9.1	172.7 ± 11.4	171.1 ± 9.8	172.1 ± 12.2	174.4 ± 9.4
BMI, kg/m^2					
Mean ± SD	27.3 ± 3.7	26.9 ± 4.0	25.6 ± 4.2	24.6 ± 3.3	26.7 ± 5.0

Randomly assigned participants who received both doses of the study vaccination and had blood collected for cell-mediated immunity testing were included in the analysis.
BMI, body mass index; IQR, interquartile range; SD, standard deviation.

Table S2. S-Specific CD4+ and CD8+ T cell response rates

	mRNA-1273	mRNA-1283			Placebo + mRNA-1283
	100 µg (n = 19)	10 µg (n = 19)	30 µg (n = 20)	100 µg (n = 21)	100 µg (n = 15)
**CD4+ T cell response rate, n/N (%)**					
**Th1**					
S1 peptide pool					
Baseline (pre-dose 1)	1/19 (5.3)	1/18 (5.6)	2/20 (10.0)	0/21 (0.0)	1/14 (7.1)
Day 36 (7 days post dose 2)	18/19 (94.7)	16/16 (100)	20/20 (100)	19/20 (95.0)	3/14 (21.4)
Day 57 (28 days post dose 2)	18/18 (100)	15/15 (100)	19/19 (100)	18/18 (100)	10/12 (83.3)
Day 209 (6 months post dose 2)	18/18 (100)	15/15 (100)	17/19 (89.5)	17/18 (94.4)	6/11 (54.5)
S2 peptide pool					
Baseline (pre-dose 1)	1/18 (5.6)	2/18 (11.1)	5/19 (26.3)	1/20 (5.0)	1/14 (7.1)
Day 36 (7 days post dose 2)	19/19 (100)	4/16 (25.0)	9/17 (52.9)	9/20 (45.0)	1/14 (7.1)
Day 57 (28 days post dose 2)	18/18 (100)	6/15 (40.0)	8/19 (42.1)	5/18 (27.8)	4/11 (36.4)
Day 209 (6 months post dose 2)	17/18 (94.4)	4/15 (26.7)	6/19 (31.6)	3/17 (17.6)	3/11 (27.3)
S1 + S2 peptide pool					
Baseline (pre-dose 1)	1/19 (5.3)	2/18 (11.1)	5/20 (25.0)	1/21 (4.8)	1/14 (7.1)
Day 36 (7 days post dose 2)	19/19 (100)	16/16 (100)	20/20 (100)	19/20 (95.0)	3/14 (21.4)
Day 57 (28 days post dose 2)	18/18 (100)	15/15 (100)	19/19 (100)	18/18 (100)	10/12 (83.3)
Day 209 (6 months post dose 2)	18/18 (100)	15/15 (100)	17/19 (89.5)	17/18 (94.4)	6/11 (54.5)
**Th2**					
S1 peptide pool					

Baseline (pre-dose 1)	0/19 (0.0)	0/18 (0.0)	0/20 (0.0)	0/21 (0.0)	0/14 (0.0)
Day 36 (7 days post dose 2)	5/19 (26.3)	2/16 (12.5)	4/20 (20.0)	3/20 (15.0)	1/14 (7.1)
Day 57 (28 days post dose 2)	2/18 (11.1)	0/15 (0.0)	2/19 (10.5)	3/18 (16.7)	0/12 (0.0)
Day 209 (6 months post dose 2)	0/18 (0.0)	0/15 (0.0)	1/19 (5.3)	3/18 (16.7)	0/11 (0.0)
S2 peptide pool					
Baseline (pre-dose 1)	0/18 (0.0)	0/18 (0.0)	0/19 (0.0)	0/20 (0.0)	0/14 (0.0)
Day 36 (7 days post dose 2)	4/19 (21.1)	0/16 (0.0)	0/17 (0.0)	0/20 (0.0)	0/14 (0.0)
Day 57 (28 days post dose 2)	1/18 (5.6)	0/15 (0.0)	0/19 (0.0)	0/18 (0.0)	0/11 (0.0)
Day 209 (6 months post dose 2)	0/18 (0.0)	0/15 (0.0)	0/19 (0.0)	0/17 (0.0)	0/11 (0.0)
S1 + S2 peptide pool					
Baseline (pre-dose 1)	0/19 (0.0)	0/18 (0.0)	0/20 (0.0)	0/21 (0.0)	0/14 (0.0)
Day 36 (7 days post dose 2)	7/19 (36.8)	2/16 (12.5)	4/20 (20.0)	3/20 (15.0)	1/14 (7.1)
Day 57 (28 days post dose 2)	2/18 (11.1)	0/15 (0.0)	2/19 (10.5)	3/18 (16.7)	0/12 (0.0)
Day 209 (6 months post dose 2)	0/18 (0.0)	0/15 (0.0)	1/19 (5.3)	3/18 (16.7)	0/11 (0.0)
**CD8+ T cell response rate, n/N (%)**					
S1 peptide pool					
Baseline (pre-dose 1)	0/19 (0.0)	0/18 (0.0)	1/19 (5.3)	0/21 (0.0)	1/14 (7.1)
Day 36 (7 days post dose 2)	10/19 (52.6)	10/16 (62.5)	12/20 (60.0)	13/20 (65.0)	3/14 (21.4)
Day 57 (28 days post dose 2)	11/18 (61.1)	12/15 (80.0)	14/19 (73.7)	14/18 (77.8)	4/12 (33.3)
Day 209 (6 months post dose 2)	5/18 (27.8)	12/15 (80.0)	9/19 (47.4)	12/18 (66.7)	4/11 (36.4)
S2 peptide pool					
Baseline (pre-dose 1)	0/18 (0.0)	0/18 (0.0)	1/18 (5.6)	0/20 (0.0)	0/14 (0.0)
Day 36 (7 days post dose 2)	3/19 (15.8)	2/16 (12.5)	1/17 (5.9)	2/20 (10.0)	0/14 (0.0)
Day 57 (28 days post dose 2)	2/18 (11.1)	2/15 (13.3)	2/19 (10.5)	1/18 (5.6)	0/11 (0.0)

Day 209 (6 months post dose 2)	2/18 (11.1)	2/15 (13.3)	1/19 (5.3)	2/17 (11.8)	1/11 (9.1)
S1 + S2 peptide pool					
Baseline (pre-dose 1)	0/19 (0.0)	0/18 (0.0)	1/19 (5.3)	0/21 (0.0)	1/14 (7.1)
Day 36 (7 days post dose 2)	10/19 (52.6)	10/16 (62.5)	12/20 (60.0)	13/20 (65.0)	3/14 (21.4)
Day 57 (28 days post dose 2)	11/18 (61.1)	12/15 (80.0)	14/19 (73.7)	14/18 (77.8)	4/12 (33.3)
Day 209 (6 months post dose 2)	5/18 (27.8)	12/15 (80.0)	9/19 (47.4)	12/18 (66.7)	4/11 (36.4)

Type 1 T helper CD4+ T cell responses included interferon-γ and/or interleukin-2.

Type 2 T helper CD4+ T cell responses included IL-4, IL-5, and/or IL-13 [or all 3 cytokines] with co-expressed CD154.

Table S3. Median Magnitude of CD4+ and CD8+ T cell Responses

	mRNA-1273	mRNA-1283			Placebo + mRNA-1283
	100 µg	10 µg	30 µg	100 µg	100 µg
**CD4+ T cell median magnitude of response**					
**Th1**					
S1 peptide pool					
Baseline (pre-dose 1)	0.001	0	0.003	-0.001	0.001
Day 36 (7 days post dose 2)	0.272	0.328	0.434	0.656	0.015
Day 57 (28 days post dose 2)	0.277	0.318	0.348	0.324	0.091
Day 209 (6 months post dose 2)	0.154	0.149	0.187	0.141	0.040
S2 peptide pool					
Baseline (pre-dose 1)	0.008	0.015	0.015	0.001	0.012
Day 36 (7 days post dose 2)	0.239	0.035	0.030	0.033	0.007
Day 57 (28 days post dose 2)	0.226	0.049	0.021	0.029	0.024
Day 209 (6 months post dose 2)	0.151	0.029	0.028	0.011	0.014
S1 + S2 peptide pool					
Baseline (pre-dose 1)	0.009	0.020	0.022	0.002	0.009
Day 36 (7 days post dose 2)	0.545	0.357	0.499	0.719	0.024
Day 57 (28 days post dose 2)	0.478	0.343	0.391	0.375	0.107
Day 209 (6 months post dose 2)	0.351	0.172	0.223	0.144	0.054
**Th2**					
S1 peptide pool					
Baseline (pre-dose 1)	0	0	0	0	0

Day 36 (7 days post dose 2)	0.009	0.007	0.005	0.008	0
Day 57 (28 days post dose 2)	0.004	0.003	0.005	0.005	0
Day 209 (6 months post dose 2)	0.002	0	0.001	0.001	0
S2 peptide pool					
Baseline (pre-dose 1)	0	0	0	0	0
Day 36 (7 days post dose 2)	0.010	0	0	0	0
Day 57 (28 days post dose 2)	0.005	0	0	0	0
Day 209 (6 months post dose 2)	0.002	0	0	0	0
S1 + S2 peptide pool					
Baseline (pre-dose 1)	0	0	0	0	0
Day 36 (7 days post dose 2)	0.023	0.007	0.006	0.008	0
Day 57 (28 days post dose 2)	0.009	0.004	0.004	0.005	0.001
Day 209 (6 months post dose 2)	0.004	0.001	0.002	0.001	0
**CD8+ T cell median magnitude of response**					
S1 peptide pool					
Baseline (pre-dose 1)	-0.003	0.002	0	-0.002	0.004
Day 36 (7 days post dose 2)	0.092	0.270	0.123	0.183	0.005
Day 57 (28 days post dose 2)	0.085	0.412	0.139	0.129	0.028
Day 209 (6 months post dose 2)	0.023	0.163	0.083	0.066	0.014
S2 peptide pool					
Baseline (pre-dose 1)	-0.005	0	0	-0.001	0.003
Day 36 (7 days post dose 2)	0.020	0.009	0.003	0.002	0.001
Day 57 (28 days post dose 2)	0.014	0	0.009	0.001	0.001
Day 209 (6 months post dose 2)	0.010	0.006	0.001	0.002	0.004

S1 + S2 peptide pool					
Baseline (pre-dose 1)	-0.003	0.002	0	-0.002	0.006
Day 36 (7 days post dose 2)	0.125	0.287	0.113	0.183	0.005
Day 57 (28 days post dose 2)	0.089	0.415	0.155	0.135	0.039
Day 209 (6 months post dose 2)	0.037	0.176	0.079	0.068	0.018

IFN, interferon; IL, interleukin.

The median magnitude is based on the background-subtracted percent of cytokine-positive CD4+/CD8+ T cells out of total CD4+/CD8+ T cells. The median magnitude of response was calculated across all participants per group.

CD4+ Th1 T cell responses included IFN-γ, IL-2, or both.

CD4+ Th2 T cell responses included IL-4, IL-5, and/or IL-13 (or all 3 cytokines) with co-expressed CD154.

CD8+ T cell responses included IFN-γ, IL-2, or both.

Table S4. Statistical significance of S-specific CD4+ Th1 (IFN-γ and/or IL-2) T cell responses between vaccine groups.

	Study Visit	Vaccine groups	*P*-value
**S1 + S2 Peptide Pool**	Day 36	mRNA-1273 (100 µg) vs mRNA-1283 (10 µg)	0.6588
		mRNA-1273 (100 µg) vs mRNA-1283 (30 µg)	0.4955
		mRNA-1273 (100 µg) vs mRNA-1283 (100 µg)	0.3653
		mRNA-1273 (100 µg) vs Placebo + mRNA-1283 (100 µg)	**<0.0001**
		mRNA-1283 (10 µg) vs mRNA-1283 (30 µg)	0.1889
		mRNA-1283 (10 µg) vs mRNA-1283 (100 µg)	0.0533
		mRNA-1283 (10 µg) vs Placebo + mRNA-1283 (100 µg)	**0.0001**
		mRNA-1283 (30 µg) vs mRNA-1283 (100 µg)	0.5831
		mRNA-1283 (30 µg) vs Placebo + mRNA-1283 (100 µg)	**<0.0001**
		mRNA-1283 (100 µg) vs Placebo + mRNA-1283 (100 µg)	**<0.0001**
	Day 57	mRNA-1273 (100 µg) vs mRNA-1283 (10 µg)	**0.0132**
		mRNA-1273 (100 µg) vs mRNA-1283 (30 µg)	0.1594
		mRNA-1273 (100 µg) vs mRNA-1283 (100 µg)	0.1427
		mRNA-1273 (100 µg) vs Placebo + mRNA-1283 (100 µg)	**0.0004**
		mRNA-1283 (10 µg) vs mRNA-1283 (30 µg)	0.3908
		mRNA-1283 (10 µg) vs mRNA-1283 (100 µg)	0.5087
		mRNA-1283 (10 µg) vs Placebo + mRNA-1283 (100 µg)	**0.0053**
		mRNA-1283 (30 µg) vs mRNA-1283 (100 µg)	0.7755
		mRNA-1283 (30 µg) vs Placebo + mRNA-1283 (100 µg)	**0.0031**
		mRNA-1283 (100 µg) vs Placebo + mRNA-1283 (100 µg)	**0.0037**
	Day 209	mRNA-1273 (100 µg) vs mRNA-1283 (10 µg)	**0.0364**
		mRNA-1273 (100 µg) vs mRNA-1283 (30 µg)	0.0919

		mRNA-1273 (100 µg) vs mRNA-1283 (100 µg)	**0.0037**
		mRNA-1273 (100 µg) vs Placebo + mRNA-1283 (100 µg)	**0.0006**
		mRNA-1283 (10 µg) vs mRNA-1283 (30 µg)	0.4505
		mRNA-1283 (10 µg) vs mRNA-1283 (100 µg)	0.5800
		mRNA-1283 (10 µg) vs Placebo + mRNA-1283 (100 µg)	**0.0150**
		mRNA-1283 (30 µg) vs mRNA-1283 (100 µg)	0.2100
		mRNA-1283 (30 µg) vs Placebo + mRNA-1283 (100 µg)	**0.0234**
		mRNA-1283 (100 µg) vs Placebo + mRNA-1283 (100 µg)	**0.0243**
**S1 Peptide Pool**	Day 36	mRNA-1273 (100 µg) vs mRNA-1283 (10 µg)	0.1090
		mRNA-1273 (100 µg) vs mRNA-1283 (30 µg)	**0.0026**
		mRNA-1273 (100 µg) vs mRNA-1283 (100 µg)	**0.0029**
		mRNA-1273 (100 µg) vs Placebo + mRNA-1283 (100 µg)	**0.0002**
		mRNA-1283 (10 µg) vs mRNA-1283 (30 µg)	0.1783
		mRNA-1283 (10 µg) vs mRNA-1283 (100 µg)	0.0575
		mRNA-1283 (10 µg) vs Placebo + mRNA-1283 (100 µg)	**0.0001**
		mRNA-1283 (30 µg) vs mRNA-1283 (100 µg)	0.5648
		mRNA-1283 (30 µg) vs Placebo + mRNA-1283 (100 µg)	**<0.0001**
		mRNA-1283 (100 µg) vs Placebo + mRNA-1283 (100 µg)	**<0.0001**
	Day 57	mRNA-1273 (100 µg) vs mRNA-1283 (10 µg)	0.3807
		mRNA-1273 (100 µg) vs mRNA-1283 (30 µg)	0.1419
		mRNA-1273 (100 µg) vs mRNA-1283 (100 µg)	0.1108
		mRNA-1273 (100 µg) vs Placebo + mRNA-1283 (100 µg)	**0.0193**
		mRNA-1283 (10 µg) vs mRNA-1283 (30 µg)	0.3720
		mRNA-1283 (10 µg) vs mRNA-1283 (100 µg)	0.4211
		mRNA-1283 (10 µg) vs Placebo + mRNA-1283 (100 µg)	**0.0031**
		mRNA-1283 (30 µg) vs mRNA-1283 (100 µg)	0.9880
		mRNA-1283 (30 µg) vs Placebo + mRNA-1283 (100 µg)	**0.0012**
		mRNA-1283 (100 µg) vs Placebo + mRNA-1283 (100 µg)	**0.0011**

	Day 209	mRNA-1273 (100 µg) vs mRNA-1283 (10 µg)	0.7347
		mRNA-1273 (100 µg) vs mRNA-1283 (30 µg)	0.8453
		mRNA-1273 (100 µg) vs mRNA-1283 (100 µg)	0.7193
		mRNA-1273 (100 µg) vs Placebo + mRNA-1283 (100 µg)	**0.0108**
		mRNA-1283 (10 µg) vs mRNA-1283 (30 µg)	0.4929
		mRNA-1283 (10 µg) vs mRNA-1283 (100 µg)	0.7080
		mRNA-1283 (10 µg) vs Placebo + mRNA-1283 (100 µg)	**0.0108**
		mRNA-1283 (30 µg) vs mRNA-1283 (100 µg)	0.3909
		mRNA-1283 (30 µg) vs Placebo + mRNA-1283 (100 µg)	**0.0161**
		mRNA-1283 (100 µg) vs Placebo + mRNA-1283 (100 µg)	**0.0081**
**S2 Peptide Pool**	Day 36	mRNA-1273 (100 µg) vs mRNA-1283 (10 µg)	**<0.0001**
		mRNA-1273 (100 µg) vs mRNA-1283 (30 µg)	**<0.0001**
		mRNA-1273 (100 µg) vs mRNA-1283 (100 µg)	**<0.0001**
		mRNA-1273 (100 µg) vs Placebo + mRNA-1283 (100 µg)	**<0.0001**
		mRNA-1283 (10 µg) vs mRNA-1283 (30 µg)	0.2922
		mRNA-1283 (10 µg) vs mRNA-1283 (100 µg)	0.6483
		mRNA-1283 (10 µg) vs Placebo + mRNA-1283 (100 µg)	**0.0472**
		mRNA-1283 (30 µg) vs mRNA-1283 (100 µg)	0.5980
		mRNA-1283 (30 µg) vs Placebo + mRNA-1283 (100 µg)	**0.0026**
		mRNA-1283 (100 µg) vs Placebo + mRNA-1283 (100 µg)	**0.0248**
	Day 57	mRNA-1273 (100 µg) vs mRNA-1283 (10 µg)	**<0.0001**
		mRNA-1273 (100 µg) vs mRNA-1283 (30 µg)	**<0.0001**
		mRNA-1273 (100 µg) vs mRNA-1283 (100 µg)	**<0.0001**
		mRNA-1273 (100 µg) vs Placebo + mRNA-1283 (100 µg)	**0.0001**
		mRNA-1283 (10 µg) vs mRNA-1283 (30 µg)	0.8911
		mRNA-1283 (10 µg) vs mRNA-1283 (100 µg)	0.1552
		mRNA-1283 (10 µg) vs Placebo + mRNA-1283 (100 µg)	0.2586
		mRNA-1283 (30 µg) vs mRNA-1283 (100 µg)	0.3909

	mRNA-1283 (30 µg) vs Placebo + mRNA-1283 (100 µg)	0.6719
	mRNA-1283 (100 µg) vs Placebo + mRNA-1283 (100 µg)	0.9472
Day 209	mRNA-1273 (100 µg) vs mRNA-1283 (10 µg)	**<0.0001**
	mRNA-1273 (100 µg) vs mRNA-1283 (30 µg)	**<0.0001**
	mRNA-1273 (100 µg) vs mRNA-1283 (100 µg)	**<0.0001**
	mRNA-1273 (100 µg) vs Placebo + mRNA-1283 (100 µg)	**<0.0001**
	mRNA-1283 (10 µg) vs mRNA-1283 (30 µg)	0.7065
	mRNA-1283 (10 µg) vs mRNA-1283 (100 µg)	0.1425
	mRNA-1283 (30 µg) vs Placebo + mRNA-1283 (100 µg)	0.3842
	mRNA-1283 (30 µg) vs mRNA-1283 (100 µg)	**0.0194**
	mRNA-1283 (30 µg) vs Placebo + mRNA-1283 (100 µg)	0.1226
	mRNA-1283 (100 µg) vs Placebo + mRNA-1283 (100 µg)	0.4869

IFN, interferon; IL, interleukin; S, spike.

P-values were calculated by Wilcoxon rank sum test. *P*-values in bold were <0.05.

Table S5. Statistical significance of S-specific CD4+ Th2 (CD4+ IL-4 and/or IL-5 and/or IL-13 and CD154) T cell responses between vaccine groups.

	Study Visit	Vaccine groups	*P*-value
**S1 + S2 Peptide Pool**	**Day 36**	mRNA-1273 (100 µg) vs mRNA-1283 (10 µg)	**0.0343**
		mRNA-1273 (100 µg) vs mRNA-1283 (30 µg)	0.0840
		mRNA-1273 (100 µg) vs mRNA-1283 (100 µg)	0.1032
		mRNA-1273 (100 µg) vs Placebo + mRNA-1283 (100 µg)	**0.0006**
		mRNA-1283 (10 µg) vs mRNA-1283 (30 µg)	1.0000
		mRNA-1283 (10 µg) vs mRNA-1283 (100 µg)	0.6483
		mRNA-1283 (10 µg) vs Placebo + mRNA-1283 (100 µg)	**0.0004**
		mRNA-1283 (30 µg) vs mRNA-1283 (100 µg)	0.3408
		mRNA-1283 (30 µg) vs Placebo + mRNA-1283 (100 µg)	**<0.0001**
		mRNA-1283 (100 µg) vs Placebo + mRNA-1283 (100 µg)	**0.0001**
	**Day 57**	mRNA-1273 (100 µg) vs mRNA-1283 (10 µg)	**0.0301**
		mRNA-1273 (100 µg) vs mRNA-1283 (30 µg)	0.2073
		mRNA-1273 (100 µg) vs mRNA-1283 (100 µg)	0.3888
		mRNA-1273 (100 µg) vs Placebo + mRNA-1283 (100 µg)	**0.0004**
		mRNA-1283 (10 µg) vs mRNA-1283 (30 µg)	0.5098
		mRNA-1283 (10 µg) vs mRNA-1283 (100 µg)	0.1776
		mRNA-1283 (10 µg) vs Placebo + mRNA-1283 (100 µg)	**0.0058**
		mRNA-1283 (30 µg) vs mRNA-1283 (100 µg)	0.4566
		mRNA-1283 (30 µg) vs Placebo + mRNA-1283 (100 µg)	**0.0088**
		mRNA-1283 (100 µg) vs Placebo + mRNA-1283 (100 µg)	**0.0001**
	**Day 209**	mRNA-1273 (100 µg) vs mRNA-1283 (10 µg)	**0.0373**
		mRNA-1273 (100 µg) vs mRNA-1283 (30 µg)	0.1754
		mRNA-1273 (100 µg) vs mRNA-1283 (100 µg)	0.5161

		mRNA-1273 (100 µg) vs Placebo + mRNA-1283 (100 µg)	**0.0133**
		mRNA-1283 (10 µg) vs mRNA-1283 (30 µg)	0.5081
		mRNA-1283 (10 µg) vs mRNA-1283 (100 µg)	0.2176
		mRNA-1283 (10 µg) vs Placebo + mRNA-1283 (100 µg)	0.2436
		mRNA-1283 (30 µg) vs mRNA-1283 (100 µg)	0.7489
		mRNA-1283 (30 µg) vs Placebo + mRNA-1283 (100 µg)	0.1144
		mRNA-1283 (100 µg) vs Placebo + mRNA-1283 (100 µg)	0.0583
**S1 Peptide Pool**	**Day 36**	mRNA-1273 (100 µg) vs mRNA-1283 (10 µg)	0.5232
		mRNA-1273 (100 µg) vs mRNA-1283 (30 µg)	0.7705
		mRNA-1273 (100 µg) vs mRNA-1283 (100 µg)	0.8221
		mRNA-1273 (100 µg) vs Placebo + mRNA-1283 (100 µg)	**0.0015**
		mRNA-1283 (10 µg) vs mRNA-1283 (30 µg)	0.8136
		mRNA-1283 (10 µg) vs mRNA-1283 (100 µg)	0.5604
		mRNA-1283 (10 µg) vs Placebo + mRNA-1283 (100 µg)	**0.0003**
		mRNA-1283 (30 µg) vs mRNA-1283 (100 µg)	0.4135
		mRNA-1283 (30 µg) vs Placebo + mRNA-1283 (100 µg)	**<0.0001**
		mRNA-1283 (100 µg) vs Placebo + mRNA-1283 (100 µg)	**<0.0001**
	**Day 57**	mRNA-1273 (100 µg) vs mRNA-1283 (10 µg)	0.6300
		mRNA-1273 (100 µg) vs mRNA-1283 (30 µg)	0.9879
		mRNA-1273 (100 µg) vs mRNA-1283 (100 µg)	0.3888
		mRNA-1273 (100 µg) vs Placebo + mRNA-1283 (100 µg)	**0.0010**
		mRNA-1283 (10 µg) vs mRNA-1283 (30 µg)	0.5098
		mRNA-1283 (10 µg) vs mRNA-1283 (100 µg)	0.1776
		mRNA-1283 (10 µg) vs Placebo + mRNA-1283 (100 µg)	**0.0026**
		mRNA-1283 (30 µg) vs mRNA-1283 (100 µg)	0.5333
		mRNA-1283 (30 µg) vs Placebo + mRNA-1283 (100 µg)	**0.0028**
		mRNA-1283 (100 µg) vs Placebo + mRNA-1283 (100 µg)	**0.0001**
	**Day 209**	mRNA-1273 (100 µg) vs mRNA-1283 (10 µg)	0.0614

		mRNA-1273 (100 µg) vs mRNA-1283 (30 µg)	0.5617
		mRNA-1273 (100 µg) vs mRNA-1283 (100 µg)	0.9873
		mRNA-1273 (100 µg) vs Placebo + mRNA-1283 (100 µg)	**0.0218**
		mRNA-1283 (10 µg) vs mRNA-1283 (30 µg)	0.2480
		mRNA-1283 (10 µg) vs mRNA-1283 (100 µg)	0.1132
		mRNA-1283 (10 µg) vs Placebo + mRNA-1283 (100 µg)	0.4498
		mRNA-1283 (30 µg) vs mRNA-1283 (100 µg)	0.7479
		mRNA-1283 (30 µg) vs Placebo + mRNA-1283 (100 µg)	0.1045
		mRNA-1283 (100 µg) vs Placebo + mRNA-1283 (100 µg)	0.0551
**S2 Peptide Pool**	**Day 36**	mRNA-1273 (100 µg) vs mRNA-1283 (10 µg)	**0.0013**
		mRNA-1273 (100 µg) vs mRNA-1283 (30 µg)	**0.0006**
		mRNA-1273 (100 µg) vs mRNA-1283 (100 µg)	**0.0004**
		mRNA-1273 (100 µg) vs Placebo + mRNA-1283 (100 µg)	**0.0006**
		mRNA-1283 (10 µg) vs mRNA-1283 (30 µg)	0.6795
		mRNA-1283 (10 µg) vs mRNA-1283 (100 µg)	0.3747
		mRNA-1283 (10 µg) vs Placebo + mRNA-1283 (100 µg)	0.7603
		mRNA-1283 (30 µg) vs mRNA-1283 (100 µg)	0.2011
		mRNA-1283 (30 µg) vs Placebo + mRNA-1283 (100 µg)	0.5640
		mRNA-1283 (100 µg) vs Placebo + mRNA-1283 (100 µg)	0.4927
	**Day 57**	mRNA-1273 (100 µg) vs mRNA-1283 (10 µg)	**0.0004**
		mRNA-1273 (100 µg) vs mRNA-1283 (30 µg)	**0.0005**
		mRNA-1273 (100 µg) vs mRNA-1283 (100 µg)	**0.0021**
		mRNA-1273 (100 µg) vs Placebo + mRNA-1283 (100 µg)	**0.0031**
		mRNA-1283 (10 µg) vs mRNA-1283 (30 µg)	0.8657
		mRNA-1283 (10 µg) vs mRNA-1283 (100 µg)	0.2675
		mRNA-1283 (10 µg) vs Placebo + mRNA-1283 (100 µg)	0.7873
		mRNA-1283 (30 µg) vs mRNA-1283 (100 µg)	0.4103
		mRNA-1283 (30 µg) vs Placebo + mRNA-1283 (100 µg)	0.5499

	mRNA-1283 (100 µg) vs Placebo + mRNA-1283 (100 µg)	0.2062
**Day 209**	mRNA-1273 (100 µg) vs mRNA-1283 (10 µg)	**0.0487**
	mRNA-1273 (100 µg) vs mRNA-1283 (30 µg)	**0.0124**
	mRNA-1273 (100 µg) vs mRNA-1283 (100 µg)	**0.0115**
	mRNA-1273 (100 µg) vs Placebo + mRNA-1283 (100 µg)	**0.0173**
	mRNA-1283 (10 µg) vs mRNA-1283 (30 µg)	0.6252
	mRNA-1283 (10 µg) vs mRNA-1283 (100 µg)	0.9673
	mRNA-1283 (30 µg) vs Placebo + mRNA-1283 (100 µg)	0.6356
	mRNA-1283 (30 µg) vs mRNA-1283 (100 µg)	0.7316
	mRNA-1283 (30 µg) vs Placebo + mRNA-1283 (100 µg)	1.0000
	mRNA-1283 (100 µg) vs Placebo + mRNA-1283 (100 µg)	0.6310

IL, interleukin; S, spike.

P-values were calculated by Wilcoxon rank sum test. *P*-values in bold were <0.05.

Table S6. Statistical significance of S-specific CD8+ (IFN-γ and/or IL-2) T cell responses between vaccine groups.

	Study Visit	Vaccine groups	*P*-value
**S1 + S2 Peptide Pool**	**Day 36**	mRNA-1273 (100 µg) vs mRNA-1283 (10 µg)	0.1247
		mRNA-1273 (100 µg) vs mRNA-1283 (30 µg)	0.6870
		mRNA-1273 (100 µg) vs mRNA-1283 (100 µg)	0.3803
		mRNA-1273 (100 µg) vs Placebo + mRNA-1283 (100 µg)	**0.0317**
		mRNA-1283 (10 µg) vs mRNA-1283 (30 µg)	0.3521
		mRNA-1283 (10 µg) vs mRNA-1283 (100 µg)	0.2758
		mRNA-1283 (10 µg) vs Placebo + mRNA-1283 (100 µg)	**0.0003**
		mRNA-1283 (30 µg) vs mRNA-1283 (100 µg)	0.9042
		mRNA-1283 (30 µg) vs Placebo + mRNA-1283 (100 µg)	**0.0122**
		mRNA-1283 (100 µg) vs Placebo + mRNA-1283 (100 µg)	**0.0013**
	**Day 57**	mRNA-1273 (100 µg) vs mRNA-1283 (10 µg)	**0.0301**
		mRNA-1273 (100 µg) vs mRNA-1283 (30 µg)	0.1505
		mRNA-1273 (100 µg) vs mRNA-1283 (100 µg)	0.3079
		mRNA-1273 (100 µg) vs Placebo + mRNA-1283 (100 µg)	0.4395
		mRNA-1283 (10 µg) vs mRNA-1283 (30 µg)	0.2710
		mRNA-1283 (10 µg) vs mRNA-1283 (100 µg)	0.2022
		mRNA-1283 (10 µg) vs Placebo + mRNA-1283 (100 µg)	**0.0214**
		mRNA-1283 (30 µg) vs mRNA-1283 (100 µg)	0.8689
		mRNA-1283 (30 µg) vs Placebo + mRNA-1283 (100 µg)	0.0585
		mRNA-1283 (100 µg) vs Placebo + mRNA-1283 (100 µg)	0.0949
	**Day 209**	mRNA-1273 (100 µg) vs mRNA-1283 (10 µg)	**0.0247**
		mRNA-1273 (100 µg) vs mRNA-1283 (30 µg)	0.7299
		mRNA-1273 (100 µg) vs mRNA-1283 (100 µg)	0.1916
		mRNA-1273 (100 µg) vs Placebo + mRNA-1283 (100 µg)	0.4923
		mRNA-1283 (10 µg) vs mRNA-1283 (30 µg)	0.0604

		mRNA-1283 (10 µg) vs mRNA-1283 (100 µg)	0.1083
		mRNA-1283 (10 µg) vs Placebo + mRNA-1283 (100 µg)	0.0609
		mRNA-1283 (30 µg) vs mRNA-1283 (100 µg)	0.5578
		mRNA-1283 (30 µg) vs Placebo + mRNA-1283 (100 µg)	0.4197
		mRNA-1283 (100 µg) vs Placebo + mRNA-1283 (100 µg)	0.1338
**S1 Peptide Pool**	**Day 36**	mRNA-1273 (100 µg) vs mRNA-1283 (10 µg)	0.0562
		mRNA-1273 (100 µg) vs mRNA-1283 (30 µg)	0.3363
		mRNA-1273 (100 µg) vs mRNA-1283 (100 µg)	0.1661
		mRNA-1273 (100 µg) vs Placebo + mRNA-1283 (100 µg)	**0.0349**
		mRNA-1283 (10 µg) vs mRNA-1283 (30 µg)	0.3521
		mRNA-1283 (10 µg) vs mRNA-1283 (100 µg)	0.4216
		mRNA-1283 (10 µg) vs Placebo + mRNA-1283 (100 µg)	**0.0002**
		mRNA-1283 (30 µg) vs mRNA-1283 (100 µg)	0.9680
		mRNA-1283 (30 µg) vs Placebo + mRNA-1283 (100 µg)	**0.0015**
		mRNA-1283 (100 µg) vs Placebo + mRNA-1283 (100 µg)	**0.0004**
	**Day 57**	mRNA-1273 (100 µg) vs mRNA-1283 (10 µg)	**0.0147**
		mRNA-1273 (100 µg) vs mRNA-1283 (30 µg)	0.0569
		mRNA-1273 (100 µg) vs mRNA-1283 (100 µg)	0.1341
		mRNA-1273 (100 µg) vs Placebo + mRNA-1283 (100 µg)	0.9502
		mRNA-1283 (10 µg) vs mRNA-1283 (30 µg)	0.2864
		mRNA-1283 (10 µg) vs mRNA-1283 (100 µg)	0.2154
		mRNA-1283 (10 µg) vs Placebo + mRNA-1283 (100 µg)	**0.0139**
		mRNA-1283 (30 µg) vs mRNA-1283 (100 µg)	0.7755
		mRNA-1283 (30 µg) vs Placebo + mRNA-1283 (100 µg)	**0.0435**
		mRNA-1283 (100 µg) vs Placebo + mRNA-1283 (100 µg)	0.1038
	**Day 209**	mRNA-1273 (100 µg) vs mRNA-1283 (10 µg)	**0.0164**
		mRNA-1273 (100 µg) vs mRNA-1283 (30 µg)	0.2579
		mRNA-1273 (100 µg) vs mRNA-1283 (100 µg)	0.0591

		mRNA-1273 (100 µg) vs Placebo + mRNA-1283 (100 µg)	0.7402
		mRNA-1283 (10 µg) vs mRNA-1283 (30 µg)	0.0604
		mRNA-1283 (10 µg) vs mRNA-1283 (100 µg)	0.1349
		mRNA-1283 (10 µg) vs Placebo + mRNA-1283 (100 µg)	**0.0362**
		mRNA-1283 (30 µg) vs mRNA-1283 (100 µg)	0.6201
		mRNA-1283 (30 µg) vs Placebo + mRNA-1283 (100 µg)	0.3279
		mRNA-1283 (100 µg) vs Placebo + mRNA-1283 (100 µg)	0.0756
**S2 Peptide Pool**	**Day 36**	mRNA-1273 (100 µg) vs mRNA-1283 (10 µg)	0.0948
		mRNA-1273 (100 µg) vs mRNA-1283 (30 µg)	**0.0110**
		mRNA-1273 (100 µg) vs mRNA-1283 (100 µg)	**0.0047**
		mRNA-1273 (100 µg) vs Placebo + mRNA-1283 (100 µg)	**0.0010**
		mRNA-1283 (10 µg) vs mRNA-1283 (30 µg)	0.1913
		mRNA-1283 (10 µg) vs mRNA-1283 (100 µg)	0.1491
		mRNA-1283 (10 µg) vs Placebo + mRNA-1283 (100 µg)	0.0771
		mRNA-1283 (30 µg) vs mRNA-1283 (100 µg)	0.9640
		mRNA-1283 (30 µg) vs Placebo + mRNA-1283 (100 µg)	0.8603
		mRNA-1283 (100 µg) vs Placebo + mRNA-1283 (100 µg)	0.7957
	**Day 57**	mRNA-1273 (100 µg) vs mRNA-1283 (10 µg)	0.0522
		mRNA-1273 (100 µg) vs mRNA-1283 (30 µg)	0.1113
		mRNA-1273 (100 µg) vs mRNA-1283 (100 µg)	**0.0037**
		mRNA-1273 (100 µg) vs Placebo + mRNA-1283 (100 µg)	**0.0214**
		mRNA-1283 (10 µg) vs mRNA-1283 (30 µg)	0.8642
		mRNA-1283 (10 µg) vs mRNA-1283 (100 µg)	0.4211
		mRNA-1283 (10 µg) vs Placebo + mRNA-1283 (100 µg)	0.9188
		mRNA-1283 (30 µg) vs mRNA-1283 (100 µg)	0.4079
		mRNA-1283 (30 µg) vs Placebo + mRNA-1283 (100 µg)	0.9326
		mRNA-1283 (100 µg) vs Placebo + mRNA-1283 (100 µg)	0.4923
	**Day 209**	mRNA-1273 (100 µg) vs mRNA-1283 (10 µg)	0.1776

mRNA-1273 (100 µg) vs mRNA-1283 (30 µg)	**0.0144**
mRNA-1273 (100 µg) vs mRNA-1283 (100 µg)	**0.0269**
mRNA-1273 (100 µg) vs Placebo + mRNA-1283 (100 µg)	0.0756
mRNA-1283 (10 µg) vs mRNA-1283 (30 µg)	0.1901
mRNA-1283 (10 µg) vs mRNA-1283 (100 µg)	0.4784
mRNA-1283 (30 µg) vs Placebo + mRNA-1283 (100 µg)	0.7597
mRNA-1283 (30 µg) vs mRNA-1283 (100 µg)	0.4329
mRNA-1283 (30 µg) vs Placebo + mRNA-1283 (100 µg)	0.5528
mRNA-1283 (100 µg) vs Placebo + mRNA-1283 (100 µg)	0.9631

IFN, interferon; IL, interleukin; S, spike.

P-values were calculated by Wilcoxon rank sum test. *P*-values in bold were <0.05.

Table S7. Statistical significance of COVID scores according to vaccine groups.

Study Visit	Vaccine groups	*P*-value
**Day 36**	mRNA-1273 (100 µg) vs mRNA-1283 (10 µg)	0.6058
	mRNA-1273 (100 µg) vs mRNA-1283 (30 µg)	0.4430
	mRNA-1273 (100 µg) vs mRNA-1283 (100 µg)	0.7277
	mRNA-1273 (100 µg) vs Placebo + mRNA-1283 (100 µg)	**0.0005**
	mRNA-1283 (10 µg) vs mRNA-1283 (30 µg)	0.7616
	mRNA-1283 (10 µg) vs mRNA-1283 (100 µg)	0.9003
	mRNA-1283 (10 µg) vs Placebo + mRNA-1283 (100 µg)	**0.0013**
	mRNA-1283 (30 µg) vs mRNA-1283 (100 µg)	0.8466
	mRNA-1283 (30 µg) vs Placebo + mRNA-1283 (100 µg)	**0.0012**
	mRNA-1283 (100 µg) vs Placebo + mRNA-1283 (100 µg)	**0.0014**
**Day 57**	mRNA-1273 (100 µg) vs mRNA-1283 (10 µg)	0.0805
	mRNA-1273 (100 µg) vs mRNA-1283 (30 µg)	0.0656
	mRNA-1273 (100 µg) vs mRNA-1283 (100 µg)	**0.0336**
	mRNA-1273 (100 µg) vs Placebo + mRNA-1283 (100 µg)	**0.0064**
	mRNA-1283 (10 µg) vs mRNA-1283 (30 µg)	0.3717
	mRNA-1283 (10 µg) vs mRNA-1283 (100 µg)	0.4769
	mRNA-1283 (10 µg) vs Placebo + mRNA-1283 (100 µg)	**0.0202**
	mRNA-1283 (30 µg) vs mRNA-1283 (100 µg)	0.9667
	mRNA-1283 (30 µg) vs Placebo + mRNA-1283 (100 µg)	0.1083
	mRNA-1283 (100 µg) vs Placebo + mRNA-1283 (100 µg)	0.1246

P-values were calculated by Wilcoxon rank sum test. *P*-values in bold are <0.05.

Table S8. Statistical significance of breadth of total SARS-CoV-2, S-Specific, and Non-S Specific TCRs after vaccination.

	Study visit	Vaccine groups	*P*-value
**Total SARS-CoV-2**	Day 36	mRNA-1273 (100 µg) vs mRNA-1283 (10 µg)	**0.0205**
		mRNA-1273 (100 µg) vs mRNA-1283 (30 µg)	0.2514
		mRNA-1273 (100 µg) vs mRNA-1283 (100 µg)	0.1404
		mRNA-1273 (100 µg) vs Placebo + mRNA-1283 (100 µg)	**0.0004**
		mRNA-1283 (10 µg) vs mRNA-1283 (30 µg)	0.9654
		mRNA-1283 (10 µg) vs mRNA-1283 (100 µg)	0.5868
		mRNA-1283 (10 µg) vs Placebo + mRNA-1283 (100 µg)	**0.0024**
		mRNA-1283 (30 µg) vs mRNA-1283 (100 µg)	0.8466
		mRNA-1283 (30 µg) vs Placebo + mRNA-1283 (100 µg)	**0.0055**
		mRNA-1283 (100 µg) vs Placebo + mRNA-1283 (100 µg)	**0.0011**
**S-Specific**	Day 36	mRNA-1273 (100 µg) vs mRNA-1283 (10 µg)	**0.0071**
		mRNA-1273 (100 µg) vs mRNA-1283 (30 µg)	0.1857
		mRNA-1273 (100 µg) vs mRNA-1283 (100 µg)	0.0646
		mRNA-1273 (100 µg) vs Placebo + mRNA-1283 (100 µg)	**0.0002**
		mRNA-1283 (10 µg) vs mRNA-1283 (30 µg)	0.9654
		mRNA-1283 (10 µg) vs mRNA-1283 (100 µg)	0.6258
		mRNA-1283 (10 µg) vs Placebo + mRNA-1283 (100 µg)	**0.0018**
		mRNA-1283 (30 µg) vs mRNA-1283 (100 µg)	0.5612
		mRNA-1283 (30 µg) vs Placebo + mRNA-1283 (100 µg)	**0.0076**
		mRNA-1283 (100 µg) vs Placebo + mRNA-1283 (100 µg)	**0.0007**
**Non-S-Specific**	Day 36	mRNA-1273 (100 µg) vs mRNA-1283 (10 µg)	0.0847
		mRNA-1273 (100 µg) vs mRNA-1283 (30 µg)	0.0819
		mRNA-1273 (100 µg) vs mRNA-1283 (100 µg)	0.5868
		mRNA-1273 (100 µg) vs Placebo + mRNA-1283 (100 µg)	0.3615
		mRNA-1283 (10 µg) vs mRNA-1283 (30 µg)	0.9195

		mRNA-1283 (10 µg) vsmRNA-1283 (100 µg)	0.1483
		mRNA-1283 (10 µg) vs Placebo + mRNA-1283 (100 µg)	0.4638
		mRNA-1283 (30 µg) vs mRNA-1283 (100 µg)	0.2179
		mRNA-1283 (30 µg) vs Placebo + mRNA-1283 (100 µg)	0.5869
		mRNA-1283 (100 µg) vs Placebo + mRNA-1283 (100 µg)	0.3910
**Total SARS-CoV-2**	Day 1 vs Day 36	mRNA-1273 (100 µg)	$\mathbf{1.53{\times}10^{-5}}$
		mRNA-1283 (10 µg)	$\mathbf{4.58{\times}10^{-5}}$
		mRNA-1283 (30 µg)	$\mathbf{5.72{\times}10^{-6}}$
		mRNA-1283 (100 µg)	$\mathbf{1.91{\times}10^{-6}}$
		Placebo + mRNA-1283 (100 µg)	**0.0125**
**S-Specific**	Day 1 vs Day 36	mRNA-1273 (100 µg)	$\mathbf{2.29{\times}10^{-5}}$
		mRNA-1283 (10 µg)	$\mathbf{4.58{\times}10^{-5}}$
		mRNA-1283 (30 µg)	$\mathbf{1.34{\times}10^{-5}}$
		mRNA-1283 (100 µg)	$\mathbf{1.91{\times}10^{-6}}$
		Placebo + mRNA-1283 (100 µg)	**0.0181**
**Non-S-Specific**	Day 1 vs Day 36	mRNA-1273 (100 µg)	0.1297
		mRNA-1283 (10 µg)	0.0638
		mRNA-1283 (30 µg)	**0.0215**
		mRNA-1283 (100 µg)	0.8408
		Placebo + mRNA-1283 (100 µg)	0.1070

S, spike; TCR, T cell receptor.
P-values were calculated by Wilcoxon rank sum test. *P*-values in bold are <0.05.

Table S9. Statistical significance of breadth of SARS-CoV-2 S-Specific NTD/RBD TCRs in CD4+ and CD8+ T cells or breadth of C-terminus TCRS in CD4+ and CD8+ T cells after vaccination.

	Study visit	Vaccine groups	*P*-value
**NTD/RBD CD4+**	Day 36	mRNA-1273 (100 µg) vs mRNA-1283 (10 µg)	0.93775
		mRNA-1273 (100 µg) vs mRNA-1283 (30 µg)	0.98848
		mRNA-1273 (100 µg) vs mRNA-1283 (100 µg)	0.62579
		mRNA-1273 (100 µg) vs Placebo + mRNA-1283 (100 µg)	**0.00179**
		mRNA-1283 (10 µg) vs mRNA-1283 (30 µg)	0.98848
		mRNA-1283 (10 µg) vs mRNA-1283 (100 µg)	0.62579
		mRNA-1283 (10 µg) vs Placebo + mRNA-1283 (100 µg)	**0.00206**
		mRNA-1283 (30 µg) vs mRNA-1283 (100 µg)	0.63296
		mRNA-1283 (30 µg) vs Placebo + mRNA-1283 (100 µg)	**0.00435**
		mRNA-1283 (100 µg) vs Placebo + mRNA-1283 (100 µg)	**0.00037**
**C-terminus CD4+**		mRNA-1273 (100 µg) vs mRNA-1283 (10 µg)	**0.00026**
		mRNA-1273 (100 µg) vs mRNA-1283 (30 µg)	$\mathbf{1.98 \times 10^{-6}}$
		mRNA-1273 (100 µg) vs mRNA-1283 (100 µg)	$\mathbf{4.71 \times 10^{-7}}$
		mRNA-1273 (100 µg) vs Placebo + mRNA-1283 (100 µg)	**0.00041**
		mRNA-1283 (10 µg) vs mRNA-1283 (30 µg)	0.24505
		mRNA-1283 (10 µg) vs mRNA-1283 (100 µg)	0.16824
		mRNA-1283 (10 µg) vs Placebo + mRNA-1283 (100 µg)	0.26346
		mRNA-1283 (30 µg) vs mRNA-1283 (100 µg)	0.64489
		mRNA-1283 (30 µg) vs Placebo + mRNA-1283 (100 µg)	0.72220
		mRNA-1283 (100 µg) vs Placebo + mRNA-1283 (100 µg)	0.97392
**NTD/RBD CD8+**		mRNA-1273 (100 µg) vs mRNA-1283 (10 µg)	0.15242
		mRNA-1273 (100 µg) vs mRNA-1283 (30 µg)	0.43785
		mRNA-1273 (100 µg) vs mRNA-1283 (100 µg)	0.75709
		mRNA-1273 (100 µg) vs Placebo + mRNA-1283 (100 µg)	**0.02314**

		mRNA-1283 (10 µg) vs mRNA-1283 (30 µg)	0.56255
		mRNA-1283 (10 µg) vs mRNA-1283 (100 µg)	0.26282
		mRNA-1283 (10 µg) vs Placebo + mRNA-1283 (100 µg)	0.31885
		mRNA-1283 (30 µg) vs mRNA-1283 (100 µg)	0.64620
		mRNA-1283 (30 µg) vs Placebo + mRNA-1283 (100 µg)	0.12340
		mRNA-1283 (100 µg) vs Placebo + mRNA-1283 (100 µg)	**0.04755**
**C-terminus CD8+**		mRNA-1273 (100 µg) vs mRNA-1283 (10 µg)	0.80100
		mRNA-1273 (100 µg) vs mRNA-1283 (30 µg)	0.22803
		mRNA-1273 (100 µg) vs mRNA-1283 (100 µg)	0.13609
		mRNA-1273 (100 µg) vs Placebo + mRNA-1283 (100 µg)	0.14861
		mRNA-1283 (10 µg) vs mRNA-1283 (30 µg)	0.21464
		mRNA-1283 (10 µg) vs mRNA-1283 (100 µg)	0.17748
		mRNA-1283 (10 µg) vs Placebo + mRNA-1283 (100 µg)	0.13652
		mRNA-1283 (30 µg) vs mRNA-1283 (100 µg)	0.71881
		mRNA-1283 (30 µg) vs Placebo + mRNA-1283 (100 µg)	0.70013
		mRNA-1283 (100 µg) vs Placebo + mRNA-1283 (100 µg)	1.00000
**NTD/RBD CD4+**	Day 1 vs Day 36	mRNA-1273 (100 µg)	$\mathbf{2.29\times10^{-5}}$
		mRNA-1283 (10 µg)	$\mathbf{1.53\times10^{-5}}$
		mRNA-1283 (30 µg)	$\mathbf{1.91\times10^{-6}}$
		mRNA-1283 (100 µg)	$\mathbf{1.91\times10^{-6}}$
		Placebo + mRNA-1283 (100 µg)	**0.02155**
**C-terminus CD4+**		mRNA-1273 (100 µg)	**0.00011**
		mRNA-1283 (10 µg)	0.48513
		mRNA-1283 (30 µg)	0.57129
		mRNA-1283 (100 µg)	0.42097
		Placebo + mRNA-1283 (100 µg)	0.78365
**NTD/RBD CD8+**		mRNA-1273 (100 µg)	**0.00038**
		mRNA-1283 (10 µg)	**0.00510**

	mRNA-1283 (30 μg)	**0.00133**
	mRNA-1283 (100 μg)	**0.00352**
	Placebo + mRNA-1283 (100 μg)	0.14148
**C-terminus CD8+**	mRNA-1273 (100 μg)	0.18343
	mRNA-1283 (10 μg)	0.55630
	mRNA-1283 (30 μg)	0.75977
	mRNA-1283 (100 μg)	0.67260
	Placebo + mRNA-1283 (100 μg)	0.42268

N-terminal domain; RBD, receptor binding domain; S, spike; TCR, T cell receptor.

P-values were calculated by Wilcoxon rank sum test. *P*-values in bold are <0.05

Table S10. Statistical significance of SARS-CoV-2 S-Specific T cell clonal expansion after vaccination.

**Study visit**	**Total number expanded**	***P*-value**
**Day 1 vs Day 36**	mRNA-1273 (100 µg) vs mRNA-1283 (10 µg)	0.50916
	mRNA-1273 (100 µg) vs mRNA-1283 (30 µg)	0.20604
	mRNA-1273 (100 µg) vs mRNA-1283 (100 µg)	0.05874
	mRNA-1273 (100 µg) vs Placebo + mRNA-1283 (100 µg)	**0.00361**
	mRNA-1283 (10 µg) vs mRNA-1283 (30 µg)	0.79558
	mRNA-1283 (10 µg) vs mRNA-1283 (100 µg)	0.26954
	mRNA-1283 (10 µg) vs Placebo + mRNA-1283 (100 µg)	**0.00151**
	mRNA-1283 (30 µg) vs mRNA-1283 (100 µg)	0.49452
	mRNA-1283 (30 µg) vs Placebo + mRNA-1283 (100 µg)	**0.00049**
	mRNA-1283 (100 µg) vs Placebo + mRNA-1283 (100 µg)	**0.00056**
**Study visit**	**Newly Expanded**	***P*-value**
**Day 1 vs Day 36**	mRNA-1273 (100 µg) vs mRNA-1283 (10 µg)	0.55231
	mRNA-1273 (100 µg) vs mRNA-1283 (30 µg)	0.21395
	mRNA-1273 (100 µg) vs mRNA-1283 (100 µg)	**0.02937**
	mRNA-1273 (100 µg) vs Placebo + mRNA-1283 (100 µg)	**0.00155**
	mRNA-1283 (10 µg) vs mRNA-1283 (30 µg)	0.75188
	mRNA-1283 (10 µg) vs mRNA-1283 (100 µg)	0.23458
	mRNA-1283 (10 µg) vs Placebo + mRNA-1283 (100 µg)	**0.00063**
	mRNA-1283 (30 µg) vs mRNA-1283 (100 µg)	0.37202
	mRNA-1283 (30 µg) vs Placebo + mRNA-1283 (100 µg)	**0.00015**
	mRNA-1283 (100 µg) vs Placebo + mRNA-1283 (100 µg)	**0.00018**

N-terminal domain; RBD, receptor binding domain; S, spike; TCR, T cell receptor.
P-values were calculated by Wilcoxon rank sum test. *P*-values in bold are <0.05

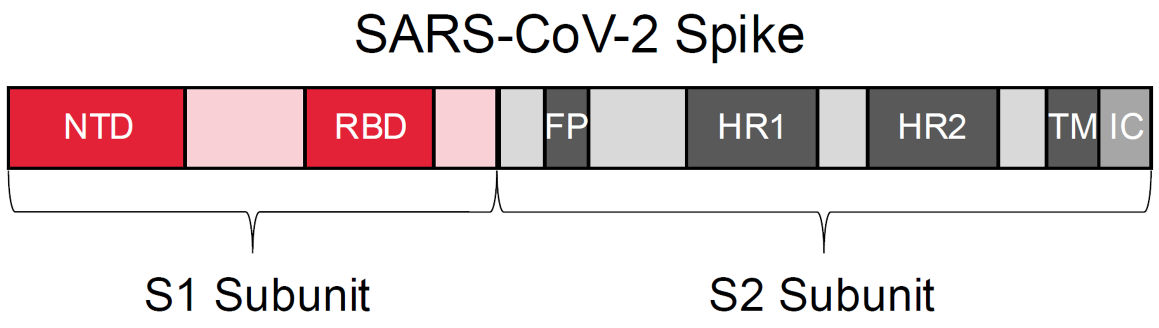

Figure S1. Schematic of SARS-CoV-2 Spike Subunits Corresponding S1/S2 Peptide Pools

Shown are the S1 and S2 subunits of the SARS-CoV-2 spike protein. The S1 peptide pool consisted primarily of RBD and NTD domains of the SARS-CoV-2 spike and are part of the mRNA-1283 vaccine construct, whereas the S2 pool comprised peptides from other domains of the D614G spike (the FP, IFP, HR1, HR2, transmembrane segment, and IC) that are absent from the mRNA-1283 vaccine construct.

FP, fusion peptide; HR, heptad repeat; IC, C-terminal domain; IFP internal fusion peptide; NTD, N-terminal domain; RBD, receptor-binding domain.

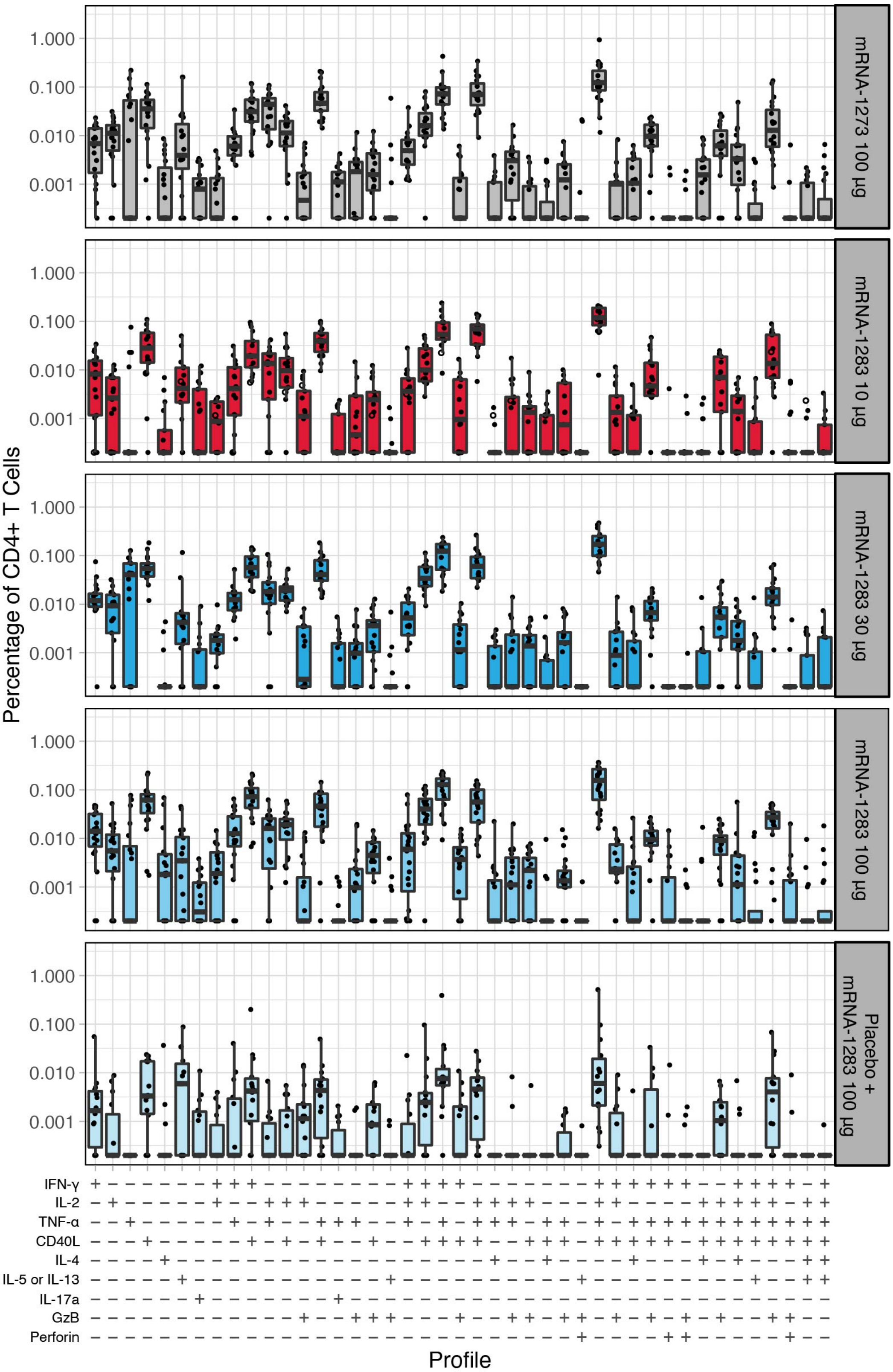

Figure S2. Polyfunctionality analysis of CD4+ T cell responses in participants in response to S1 and S2 peptide pools at Day 36.

Polyfunctionality was calculated as the frequency of the Boolean combination of cytokines detected or not detected within the total CD4+ T cell population. Percentages of cytokine expressing CD4+ T cells in response to total S (S1 and S2 peptide pools) at Day 36 for individual participant (as

represented by dots) are shown according to vaccine group. Boxes and horizontal bars denote the first and third quartiles and the medians, respectively, and whisker endpoints equal to the largest value within 1.5 times the first and third quartiles. The key below the figure shows the functional marker(s) expressed in each column, increasing in polyfunctionality to the right.
GzB, granzyme B; IFN, interferon gamma; IL, interleukin; TNF, tumour necrosis factor alpha.

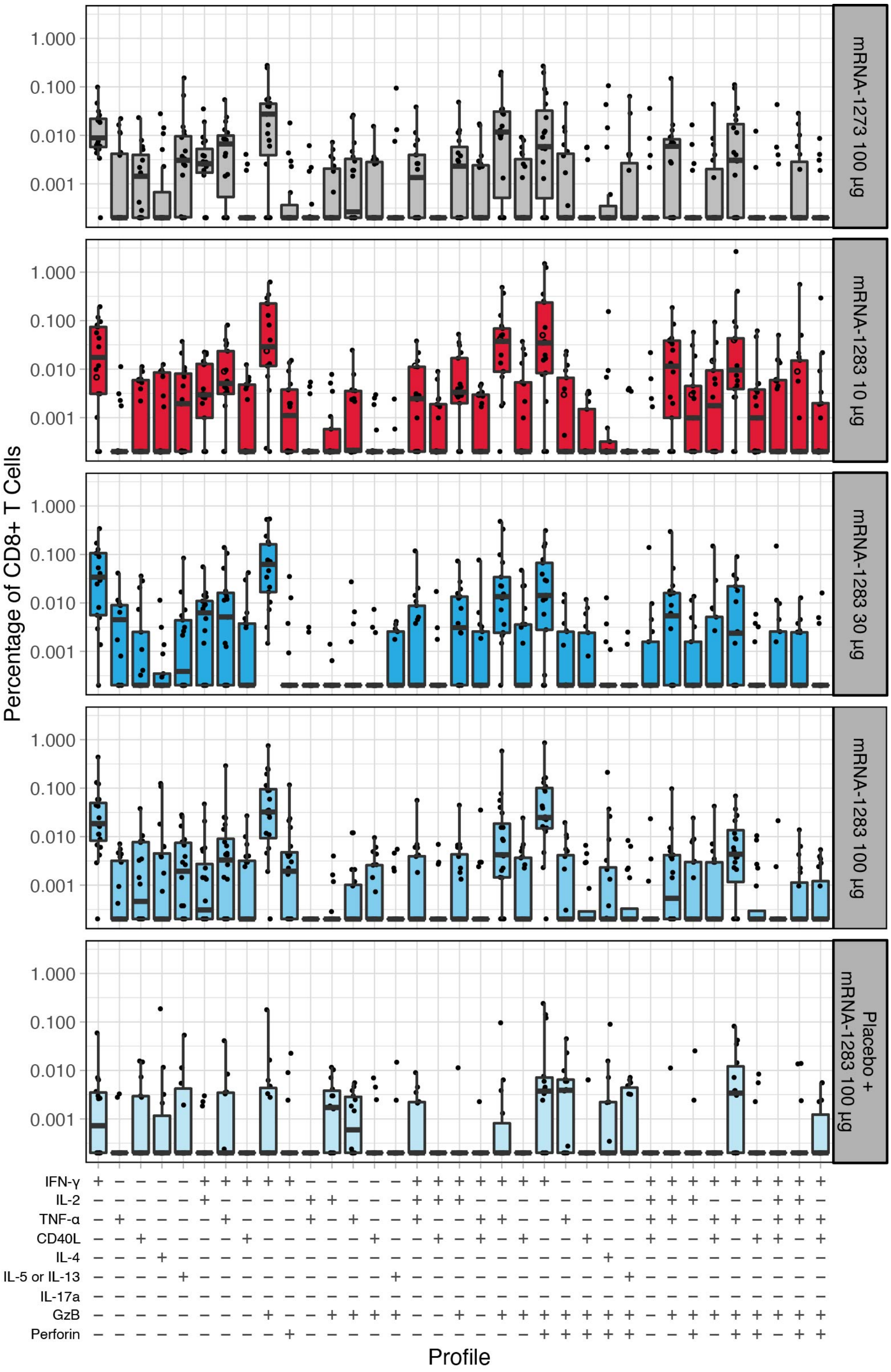

Figure S3. Polyfunctionality analysis of CD8+ T cell responses in participants in response to S1 and S2 peptide pools at Day 36.

Polyfunctionality was calculated as the frequency of the Boolean combination of cytokines detected or not detected within the total CD8+ T cell population. Percentages of cytokine expressing CD8+ T cells in response to total S (S1 and S2 peptide pools) at Day 36 for individual participant (as represented by dots) are shown according to vaccine group. Boxes and horizontal bars denote the first

and third quartiles and the medians, respectively, and whisker endpoints equal to the largest value within 1.5 times the first and third quartiles. The key below the figure shows the functional marker(s) expressed in each column, increasing in polyfunctionality to the right.
GzB, granzyme B; IFN-γ, interferon gamma; IL, interleukin; TNF-α, tumour necrosis factor alpha.

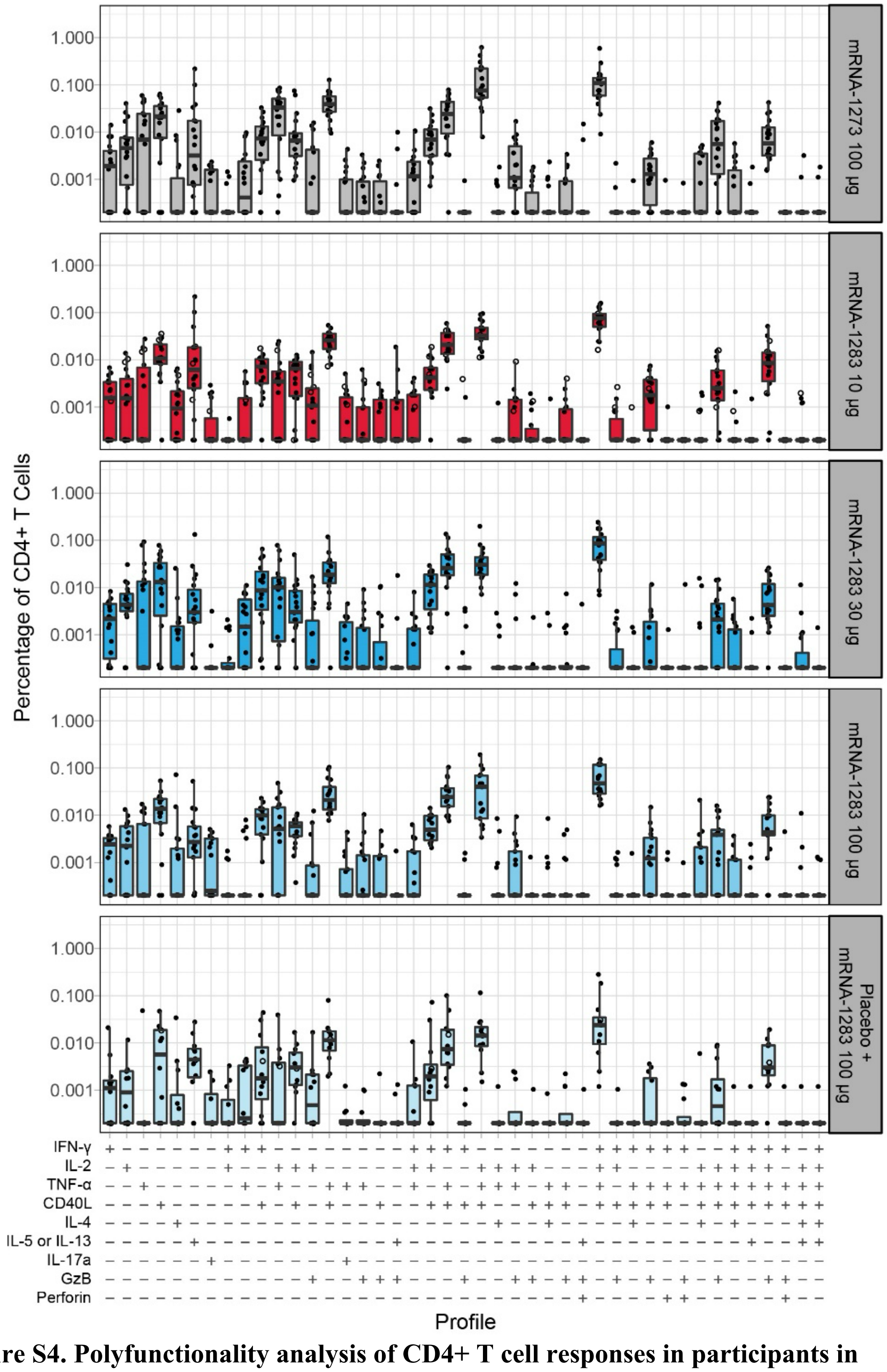

Figure S4. Polyfunctionality analysis of CD4+ T cell responses in participants in response to S1 and S2 peptide pools at Day 209.

Polyfunctionality was calculated as the frequency of the Boolean combination of cytokines detected or not detected within the total CD4+ T cell population. Percentages of cytokine expressing CD4+ T cells in response to total S (S1 and S2 peptide pools) at Day 209 for individual participant (as represented by dots) are shown according to vaccine group. Boxes and horizontal bars denote the first

and third quartiles and the medians, respectively, and whisker endpoints equal to the largest value within 1.5 times the first and third quartiles. The key below the figure shows the functional marker(s) expressed in each column, increasing in polyfunctionality to the right.
GzB, granzyme B; IFN-γ, interferon gamma; IL, interleukin; TNF-α, tumour necrosis factor alpha.

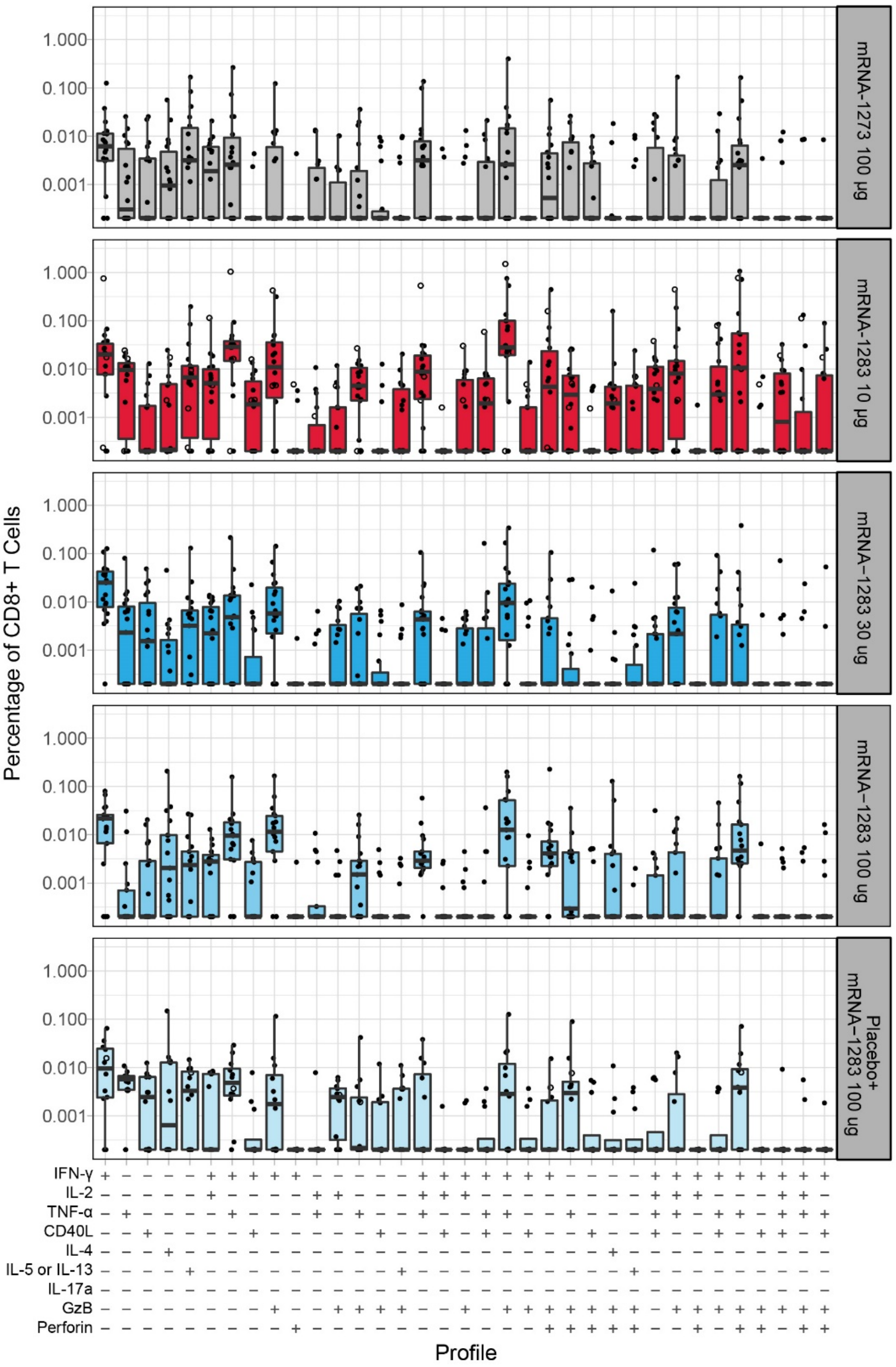

Figure S5. Polyfunctionality analysis of CD8+ T cell responses in participants in response to S1 and S2 peptide pools at Day 209.

Polyfunctionality was calculated as the frequency of the Boolean combination of cytokines detected or not detected within the total CD8+ T cell population. Percentages of cytokine expressing CD8+ T cells in response to total S (S1 and S2 peptide pools) at Day 209 for individual participant (as represented by dots) are shown according to vaccine group. Boxes and horizontal bars denote the first and third quartiles and the medians, respectively, and whisker endpoints equal to the largest value

within 1.5 times the first and third quartiles. The key below the figure shows the functional marker(s) expressed in each column, increasing in polyfunctionality to the right.
GzB, granzyme B; IFN-γ, interferon gamma; IL, interleukin; TNF-α, tumour necrosis factor alpha.

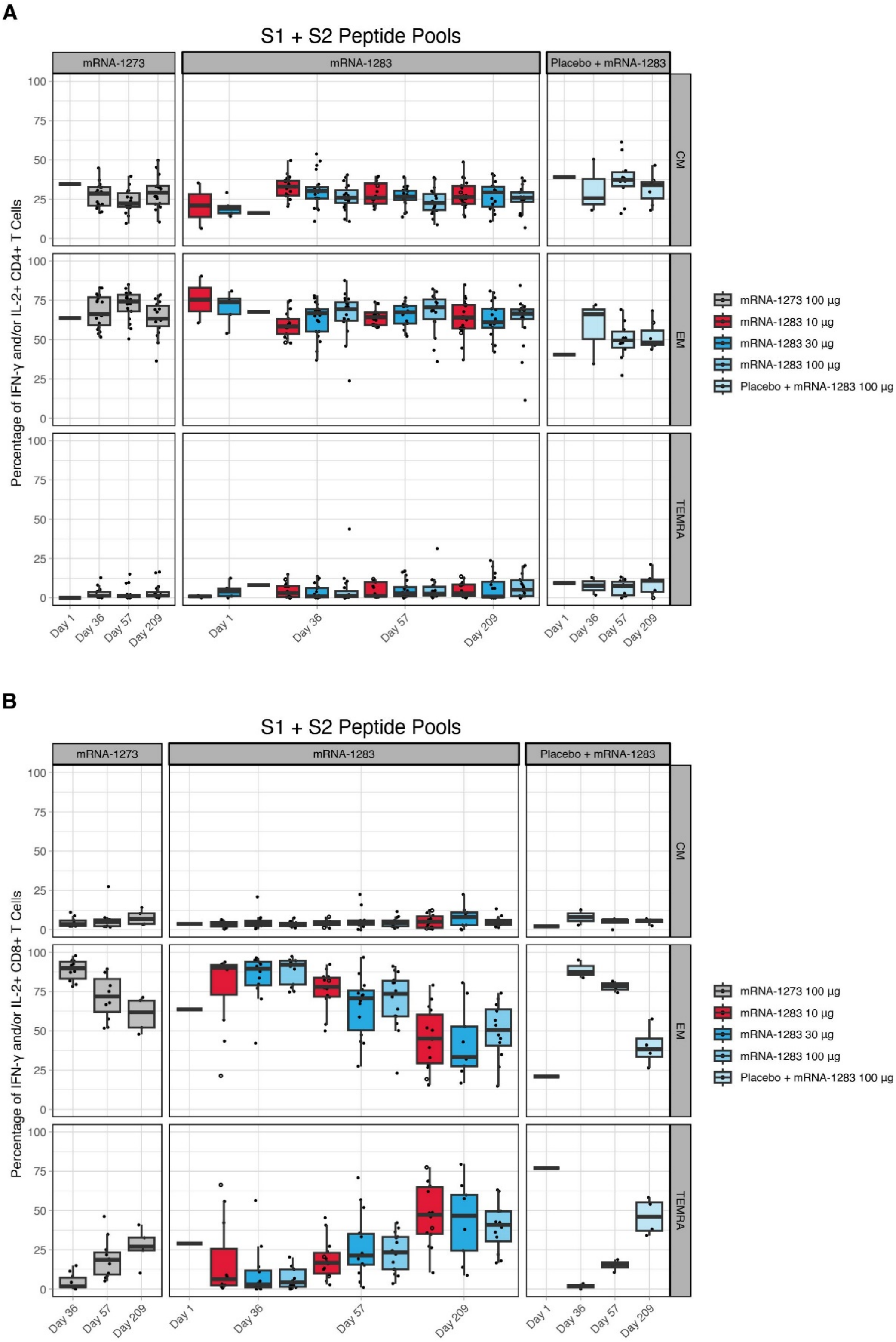

Figure S6. Longitudinal differentiation patterns of SARS-CoV-2 S-specific T cells after vaccination.

Frequencies of CD4+ (A) and CD8+ (B) memory T cells producing IFN-γ or IL-2 from cryopreserved PBMCs of mRNA-1283 or mRNA-1273 vaccine groups following *ex vivo* stimulation with SARS-CoV-2 peptides covering the total S at Visit 1 (baseline), Visit 4 (Day 36, 1 week post dose 2), Visit 5 (Day 57, 1 month post dose 2), and Visit 6 (Day 209) as measured by flow cytometry. Boxes and horizontal bars denote the first and third quartiles and the medians, respectively, and whisker

endpoints equal to the largest value within 1.5 times the first and third quartiles. Within each set of graphs, the top row shows data for CM, the middle for EM and the bottom for TEMRA cells. The percent is the proportion of the total cytokine-expressing cells (IFN-γ and/or IL-2) that are of each of these memory phenotypes; this is calculated only for positive responses.
CM, central memory cells; EM, effector memory cells; IFN-γ, interferon gamma; IL, interleukin; PBMC, peripheral blood mononuclear cell; S, spike; TEMRA, effector memory T cells re-expressing CD45RA (CD45RA+/CCR7-).

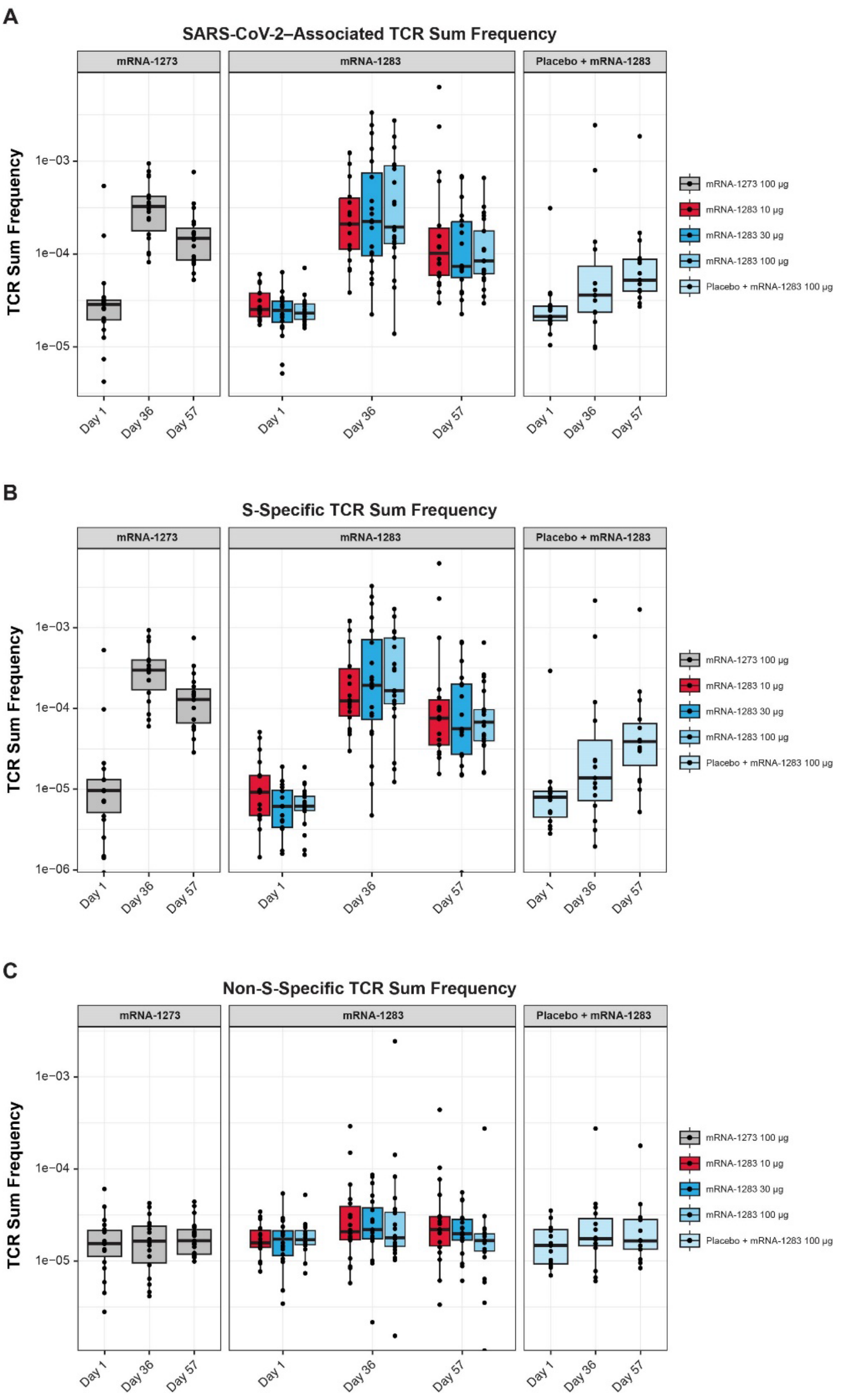

Figure S7. Sum frequency of (A) SARS-CoV-2-associated TCRs, (B) SARS-CoV-2 S-specific TCRs, and (C) non-S-specific TCRs after vaccination.

Each panel displays the number of (A) SARS-CoV-2–associated, (B) SARS-CoV-2 S-specific, or (C) non–S-specific T cells relative to the total number of T cells for each vaccine group and time point.

Individual participant repertoires (as represented by dots) are shown according to vaccine group and time point. Boxes and horizontal bars denote the first and third quartiles and the medians, respectively, and whisker endpoints equal to the largest value within 1.5 times the first and third quartiles.
TCR, T cell receptor.

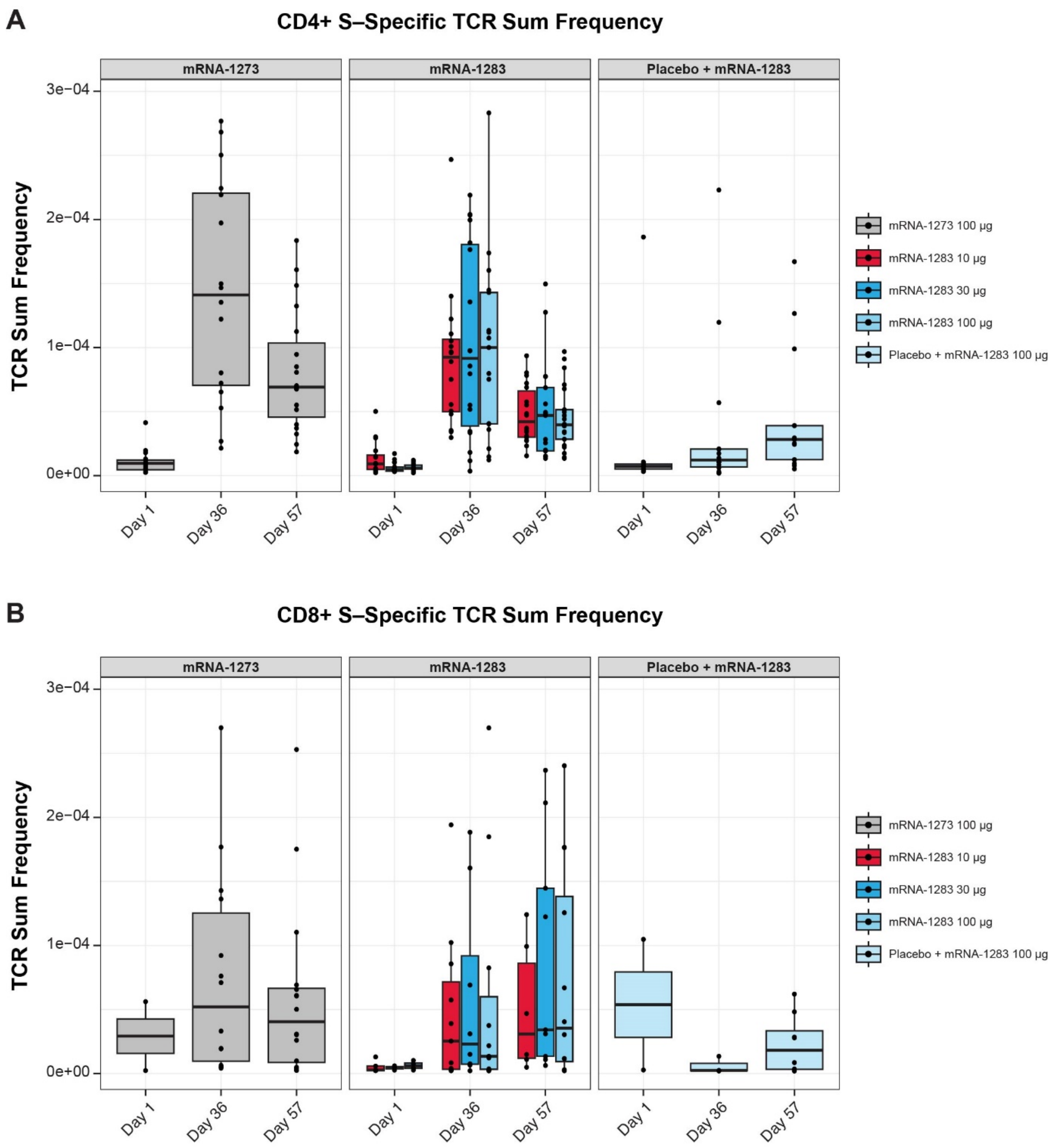

Figure S8. S-specific T cell sum frequency in (A) CD4+ and (B) CD8+ T cells after vaccination.

Each panel displays the sum frequency of S-specific TCRs for individual participants (as represented by dots) according to vaccine group and time point. Boxes and horizontal bars denote the first and third quartiles and the medians, respectively, and whisker endpoints equal to the largest value within 1.5 times the first and third quartiles.

TCR, T cell receptor.

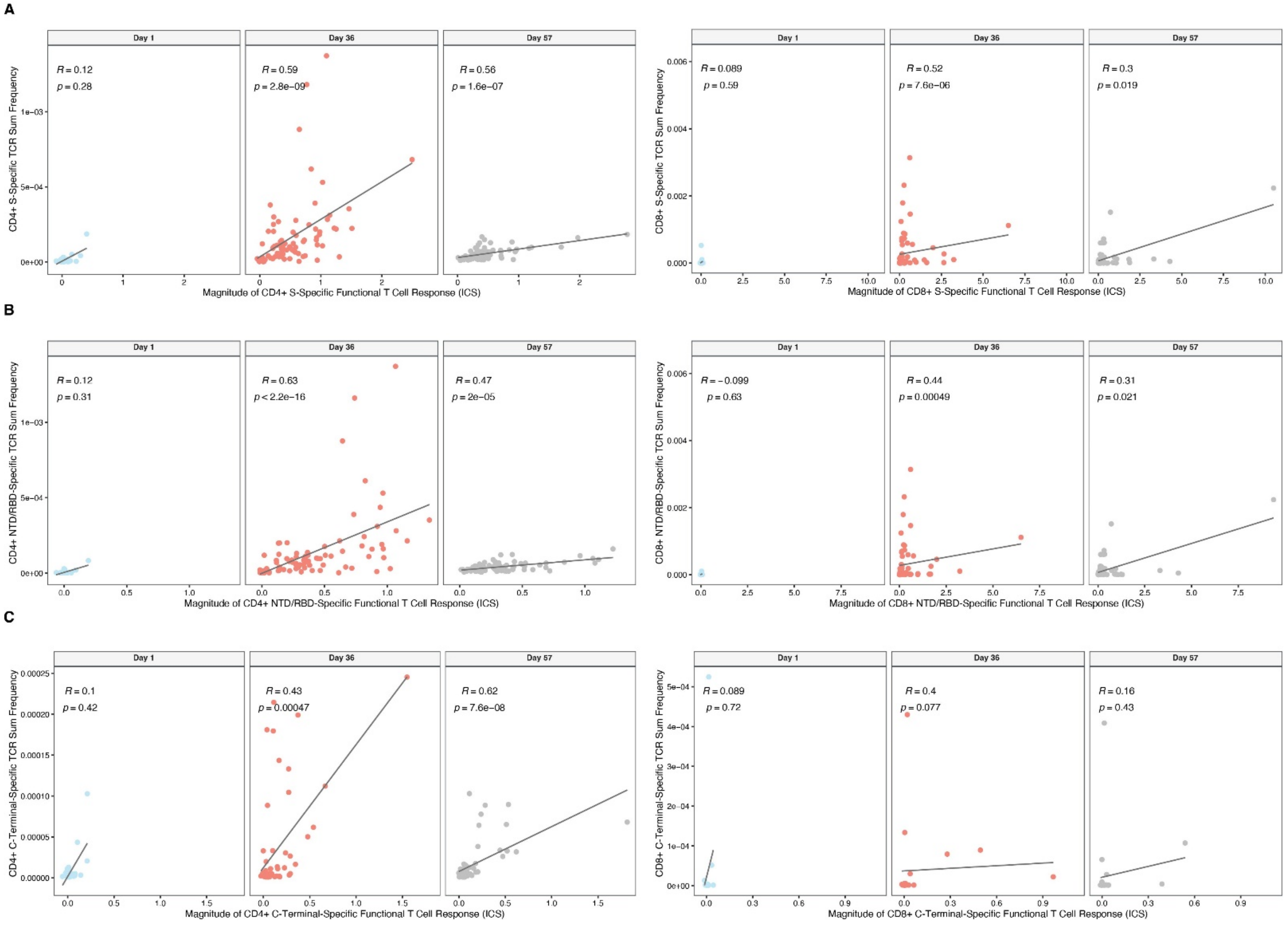

Figure S9. Correlations of S-specific TCR sum frequency with magnitude of functional T cells by ICS assay.

(A) Correlations of S-specific CD4+ and CD8+ T cell responses by sum frequency of known TCRs (y-axis) with the percentage of S-specific T cells by functional cytokine responses (IFN-γ and/or IL-2) in the intracellular cytokine staining assay (x-axis). (B) Correlations of NTD/RBD specific CD4+ and

CD8+ T cell responses by sum frequency of known TCRs (y-axis) with the percentage of T cells by functional cytokine responses in the ICS assay (x-axis). (C) Correlations of C-terminal specific CD4+ and CD8+ T cell responses by sum frequency of known TCRs (y-axis) with the percentage of T cells by functional cytokine responses in the ICS assay (x-axis). Correlation determined by Spearman rank. ICS, intracellular cytokine staining; N-terminal domain; RBD, receptor binding domain; S, spike; TCR, T cell receptor.

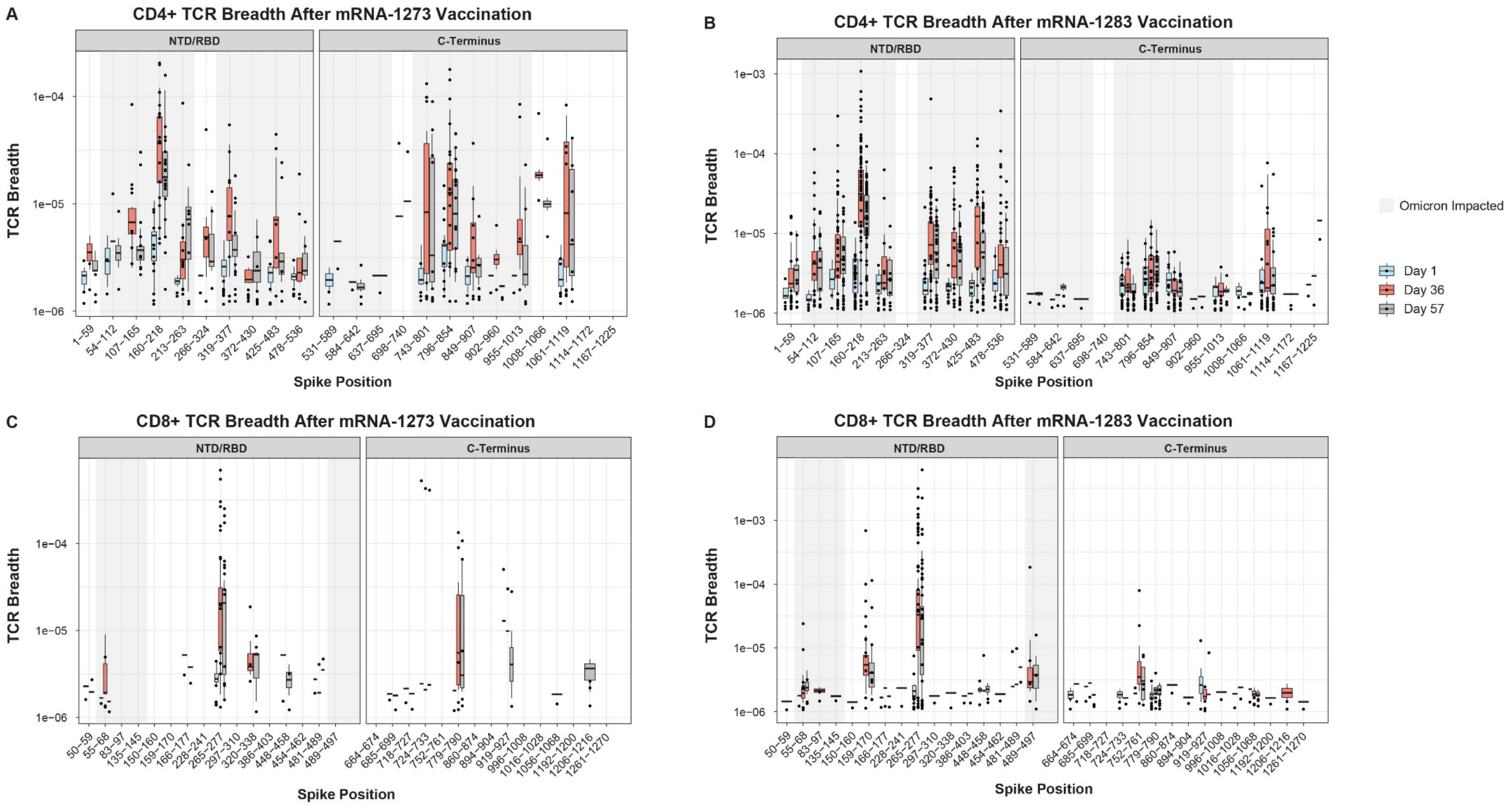

Figure S10. Breadth of CD4+ and CD8+ T cell responses mapped to omicron impacted S-protein epitopes following vaccination.

Breadth of (A, B) CD4+ and (C, D) CD8+ T cell responses after vaccination with mRNA-1273 and mRNA-1283 are shown. Epitopes are mapped to amino acid position within the S protein, with gray areas indicating overlapping regions with known omicron mutations. S, spike; TCR, T cell receptor.

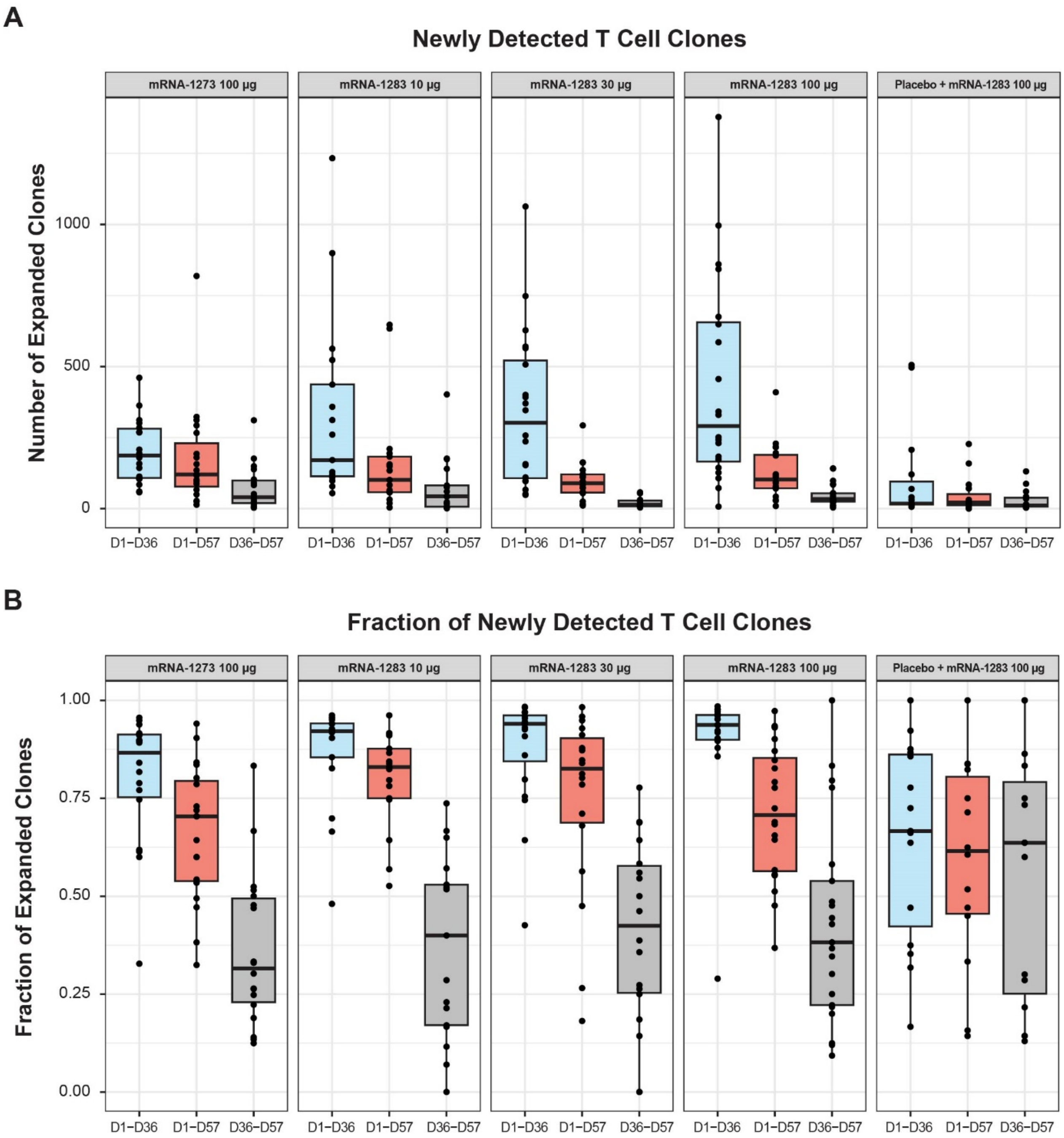

Figure S11. (A) T cell clonal expansion and (B) fraction of newly detected expanded clones after vaccination by regimen.

(A) Number of expanded clones from Day 1 to Day 36, Day 1 to Day 57, or Day 36 to Day 57, with boxes and horizontal bars denoting the first and third quartiles and the medians, respectively, and whisker endpoints equal to the largest value within 1.5 times the first and third quartiles. (B) Fraction of newly detected expanded clones from Day 1 to Day 36, Day 1 to Day 57, or Day 36 to Day 57.

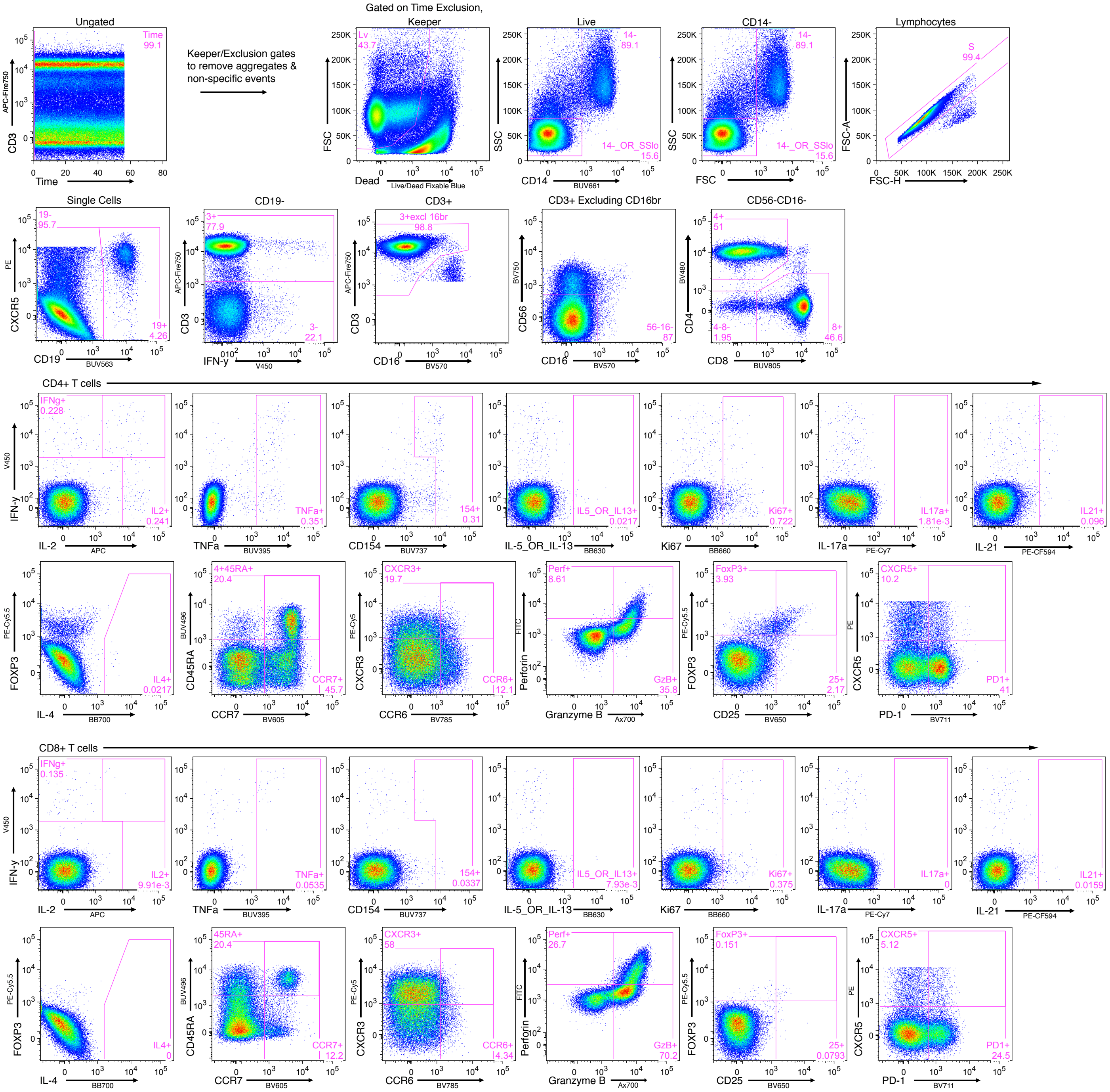

Figure S12. Representative flow cytometry gating strategy for intracellular cytokine staining assay. Representative example of ICS CD4+ and CD8+ T cell cytokine responses and gating hierarchy from a post-vaccination PBMC sample stimulated with S1 peptides.
